# Association Between Patient Factors and the Effectiveness of Wearable Trackers at Increasing the Number of Steps per Day Among Adults With Cardiometabolic Conditions: Meta-analysis of Individual Patient Data From Randomized Controlled Trials

**DOI:** 10.2196/36337

**Published:** 2022-08-30

**Authors:** Alexander Hodkinson, Evangelos Kontopantelis, Salwa S Zghebi, Christos Grigoroglou, Brian McMillan, Harm van Marwijk, Peter Bower, Dialechti Tsimpida, Charles F Emery, Mark R Burge, Hunter Esmiol, Margaret E Cupples, Mark A Tully, Kaberi Dasgupta, Stella S Daskalopoulou, Alexandra B Cooke, Ayorinde F Fayehun, Julie Houle, Paul Poirier, Thomas Yates, Joseph Henson, Derek R Anderson, Elisabeth B Grey, Maria Panagioti

**Affiliations:** 1 Division of Population Health School of Health Sciences, Faculty of Biology, Medicine and Health University of Manchester Manchester United Kingdom; 2 Health Services Research and Primary Care National Institute for Health Research School for Primary Care Research Manchester United Kingdom; 3 Division of Informatics, Imaging & Data Sciences Faculty of Biology, Medicine & Health University of Manchester Manchester United Kingdom; 4 Department of Primary Care and Public Health Brighton and Sussex Medical School University of Brighton Brighton United Kingdom; 5 Department of Psychology The Ohio State University College of Arts and Sciences Columbus, OH United States; 6 Department of Medicine, Endocrinology and Metabolism University of New Mexico Health Sciences Center Albuquerque, NM United States; 7 Department of General Practice and Primary Care Centre for Public Heath Queen’s University Belfast Belfast United Kingdom; 8 School of Medicine Ulster University Londonderry United Kingdom; 9 Department of Medicine McGill University Montreal, QC Canada; 10 Centre for Outcomes Research and Evaluation Research Institute of the McGill University Health Centre Montreal, QC Canada; 11 Centre for Translational Biology Research Institute of the McGill University Health Centre Montreal, QC Canada; 12 Department of Family Medicine University College Hospital Ibadan Nigeria; 13 Department of Nursing Université du Québec à Trois-Rivières Trois-Rivières, QC Canada; 14 Institut Universitaire de Cardiologie et de Pneumologie de Québec Université Laval Laval, QC Canada; 15 Diabetes Research Centre University of Leicester Leicester United Kingdom; 16 Centre for Motivation and Health Behaviour Change Department for Health University of Bath Bath United Kingdom

**Keywords:** systematic review, individual patient data, meta-analysis, steps/day, wearable tracker, cardiometabolic conditions, diabetes, obesity, cardiovascular disease

## Abstract

**Background:**

Current evidence supports the use of wearable trackers by people with cardiometabolic conditions. However, as the health benefits are small and confounded by heterogeneity, there remains uncertainty as to which patient groups are most helped by wearable trackers.

**Objective:**

This study examined the effects of wearable trackers in patients with cardiometabolic conditions to identify subgroups of patients who most benefited and to understand interventional differences.

**Methods:**

We obtained individual participant data from randomized controlled trials of wearable trackers that were conducted before December 2020 and measured steps per day as the primary outcome in participants with cardiometabolic conditions including diabetes, overweight or obesity, and cardiovascular disease. We used statistical models to account for clustering of participants within trials and heterogeneity across trials to estimate mean differences with the 95% CI.

**Results:**

Individual participant data were obtained from 9 of 25 eligible randomized controlled trials, which included 1481 of 3178 (47%) total participants. The wearable trackers revealed that over the median duration of 12 weeks, steps per day increased by 1656 (95% CI 918-2395), a significant change. Greater increases in steps per day from interventions using wearable trackers were observed in men (interaction coefficient –668, 95% CI –1157 to –180), patients in age categories over 50 years (50-59 years: interaction coefficient 1175, 95% CI 377-1973; 60-69 years: interaction coefficient 981, 95% CI 222-1740; 70-90 years: interaction coefficient 1060, 95% CI 200-1920), White patients (interaction coefficient 995, 95% CI 360-1631), and patients with fewer comorbidities (interaction coefficient –517, 95% CI –1188 to –11) compared to women, those aged below 50, non-White patients, and patients with multimorbidity. In terms of interventional differences, only face-to-face delivery of the tracker impacted the effectiveness of the interventions by increasing steps per day.

**Conclusions:**

In patients with cardiometabolic conditions, interventions using wearable trackers to improve steps per day mostly benefited older White men without multimorbidity.

**Trial Registration:**

PROSPERO CRD42019143012; https://www.crd.york.ac.uk/prospero/display_record.php?RecordID=143012

## Introduction

### Background

Cardiometabolic conditions are the leading cause of death worldwide, accounting for more than 41 million deaths annually [1]. These conditions include obesity, diabetes mellitus, and cardiovascular disease (CVD); these 3 common, intersecting noncommunicable diseases affect almost two-thirds of the global population [2,3].

The World Health Organization recently recognized physical inactivity as a serious and growing public health problem and has set out to reduce it by 10% before 2025 [4]. The United Kingdom’s National Institute for Health and Care Excellence also highlights the importance of physical activity for obesity management, successful aging, CVD prevention, and weight management during pregnancy [5]. The consequences of being physically inactive include unhealthy weight gain, dyslipidemia, and elevated blood pressure and blood glucose levels, all of which heighten the risk of developing a cardiometabolic condition [6].

Wearable physical activity trackers, such as accelerometers, pedometers, and the Fitbit (Fitbit Inc), are portable electrical or electromechanical devices that count each step a person takes by detecting the motion of the person along the body’s long axis [7,8]. They have become very popular for motivating and monitoring (thereby increasing) physical activity in general and in people with cardiometabolic conditions in particular [9,10]. Systematic reviews have suggested that the validity of various wearable trackers, especially those measuring steps, is high, and these reviews have found that they are useful for tracking ambulatory physical activity in clinical populations [11-13].

Since many wearable trackers are affordable and user-friendly, they are viewed as a good practical method for monitoring basic physical activity [14], such as the number of steps per day, in high-risk people with chronic cardiometabolic conditions [15-17]. However, their long-term effectiveness in achieving the desired behavior changes (ie, increasing steps per day) in specific patient subgroups with cardiometabolic conditions is unclear, and they may only succeed in the short term as “quick fixes” [18].

Our recent meta-analysis of 38 randomized trials [19] suggested that interventions using wearable trackers are moderately effective at increasing physical activity, including steps per day, in people with cardiometabolic conditions. The most promising interventions were those that focused on the number of steps per day. However, without individual participant data (IPD), we could not conduct an assessment of patient factors, baseline effects, and interventional differences, nor could we analyze their importance [20]. This was a major constraint of the review findings.

In IPD meta-analysis, rather than extracting summary (ie, aggregate) data from study publications or from investigators, original research data are obtained directly from the researchers responsible for each study. These data are then re-analyzed centrally and combined in the meta-analysis. The IPD approach is becoming an increasingly popular tool compared to traditional aggregate-data meta-analysis, especially as the IPD approach avoids reliance on published results and provides an opportunity to investigate individual-level interactions, such as treatment-effect modifiers [20].

### Objectives

In the present study, we undertook an IPD meta-analysis to identify whether belonging to certain subgroups of patients with cardiometabolic conditions, including groups with differing age, sex, ethnicity, and number or combination of cardiometabolic conditions, moderated the effectiveness of interventions using wearable trackers in improving physical activity, measured by the number of steps per day. We also examined the impact of interventional differences, such as behavior change, device placement, delivery method, and performance over time, on the effectiveness of interventions using wearable trackers in improving steps per day.

## Methods

This IPD meta-analysis followed a registered (PROSPERO CRD42019143012) protocol. A statistical analysis plan was produced in advance of analysis and the findings are reported in accordance with the PRISMA-IPD statement [21].

### Literature Search and Study Identification

We searched MEDLINE (Ovid), EMBASE (EBSCO), CENTRAL, CINAHL, and PsycINFO from inception until August 2018, without language restriction; this was updated in December 2020 (Multimedia Appendix 1, pages 3-11). Additional studies were obtained by citation tracking, extraction from previous systematic reviews, and searches of trial registers (ie, ClinicalTrials.gov and ICTRP). A list of all the search sources and the data collection and management process are detailed in the protocol for this paper.

Two researchers (AH and MP) independently identified the citations and then fully screened the relevant manuscripts according to the eligibility criteria. We included randomized controlled trials (RCTs) or cluster RCTs involving adults (aged 16 years or older) with a cardiometabolic condition, defined as a diagnosis (or high risk) of type 2 diabetes, CVD, or obesity or overweight.

We included studies with an intervention program designed around the daily usage of wearable trackers, such as pedometers, accelerometers, and fitness trackers, rather than studies that only measured performance at the beginning and end of the study. Studies were required to have a usual-care comparator. Participants’ steps per day in both intervention and usual-care groups were measured in parallel using the same device. We further required that studies reported a physical activity assessment (ie, step count) at baseline and follow up using a separate wearable tracker that all participants received independently of their allocation (ie, both the intervention and control groups). We adopted this eligibility criterion to be able to reliably pool the results across the studies.

The primary outcome was the number of steps per day, measured with any wearable tracker. Wearable trackers, either mechanical (eg, spring levered) or electronic (eg, using GPS or actigraph functionality), mostly monitored daily steps as the main outcome of interest; intervention programs involving wearable trackers often set goals for increasing the number of daily steps incrementally over time and estimated and logged the total distance travelled [22]. We excluded wearable trackers that used other types of measurement output, because these vary considerably in terms of their performance, choice of activity measurement (ie, light, moderate, or vigorous activity, energy expenditure, sedentary time, or stationary pattern) and intensity and frequency of the physical activity (ie, bouts of exercise). This would have made their pooling more problematic.

Secondary outcomes included anthropometric measures, glycated hemoglobin level (mmols/mol), blood pressure, and cholesterol level.

Risk of bias (RoB) was independently assessed by 2 reviewers (AH and MP) using the Cochrane RoB tool [23], alongside the completeness and quality of the provided IPD. Results of IPD studies were also compared with studies that did not supply IPD.

### Data Extraction and Assessment of IPD Integrity

We used IPD (obtained from November 2019 to October 2021) to determine demographic characteristics that we chose a priori in the protocol, such as age, sex, ethnicity, and comorbidity; intervention characteristics, such as objective, duration, use of a behavior change framework, delivery method and placement of wearable tracker; and primary and secondary outcomes.

Continuous variables were kept continuous, but some were also categorized when this was considered to be more clinically meaningful. For instance, patient groups were split by median age and by age range (20-49, 50-59, 60-69, and 70-90 years); the total number of comorbidities, predefined as cardiometabolic conditions including type 2 diabetes, hypertension, obesity or overweight, metabolic syndrome, and any cardiovascular condition; and BMI (normal 18.5-24.9 kg/m^2^; overweight, 25-29.9 kg/m^2^; obese, ≥30 kg/m^2^). Descriptive characteristics (eg, age, comorbidity, education, and ethnicity) were analyzed for intervention and control groups using ANOVA for continuous variables and the chi-square test for categorical variables. Following this, ethnicity was classified into 2 groups, White European/North American and other ethnicities, to improve the analysis of covariance (ANCOVA), due to the limited number of patients in other ethnic groups.

Since all the trials provided above 70% of the IPD for the corresponding primary outcome, we imputed any missing values using the R package MICE: Multivariate Imputation by Chained Equations [24], following Rubin’s principle for imputation [25]. The range of imputed values was bounded by the observed values of the primary outcome, and baseline covariates (study, intervention, age, sex, and baseline) were used to predict missing data. The algorithms’ convergence was assessed, and sensitivity analyses were performed using only cases with available data (ie, complete cases).

### Data Synthesis

Primary analyses used a 1-stage linear mixed effect model that incorporated random effects to allow for heterogeneity across trials [20,26,27], fitted using the Stata (version 16.1; StataCorp LLC) commands mixed and ipdforest to summarize the evidence by study and obtain forest plots [28]. Restricted maximum likelihood was used for model estimation, and centering of covariates by study-specific means was performed to avoid aggregation bias [29].

As the primary outcomes were all objectively measured using the standard unit of steps per day, the analysis was performed using the mean difference (MD) between intervention and control groups with the 95% CI. Differential effects were then investigated by adding patient covariate parameters (ie, age, sex, ethnicity, and number of cardiometabolic conditions) and interactions between covariates (ie, treatment-covariate interaction terms) to the linear mixed model for the primary outcome. Important and significant differential effects were displayed through subgroup analysis plots.

Results from a 2-stage random-effects (DerSimonian-Laird) meta-analysis were compared to results from 1-stage analyses using the ipdmetan command, for consistency. The Hartung-Knapp CI was used to account for uncertainty in the variance estimate [30].

Heterogeneity was examined by visually inspecting forest plots and using the *I*^2^ statistic with the 95% CI [31]. Publication bias was assessed visually by using contour-enhanced funnel plots and the statistical Egger test for asymmetry [32]. The Egger test is performed using the following hypothesis, testing by *P* value: the null hypothesis is symmetry in the funnel plot, and the alternative hypothesis is asymmetry in the funnel plot. If *P*≤.05, we reject the null hypothesis. If *P*>.05, we accept the null hypothesis and reject the alternative hypothesis [33]. We assessed for availability bias by comparing summary results of the non-IPD studies with those from IPD studies [34].

Sensitivity analyses of study-level factors were performed by (1) comparing studies that used a social cognitive theory framework as a guide for behavior change, (2) comparing studies that used a goal or goals as part of the intervention, (3) comparing the placement of the wearable tracker (ie, on the waist or wrist), (4) comparing the delivery of the intervention (ie, face to face or self-managed), (5) removing studies with high or unclear RoB based on allocation concealment, and (6) assessing the long-term performance (ie, beyond 6 months).

### Ethical Considerations

Ethical approval and patient consent was not required as it had already been obtain from the primary authors during their trial period. Ethical waiver was provided and acknowledged by our University international review board.

## Results

**Figure 1:**
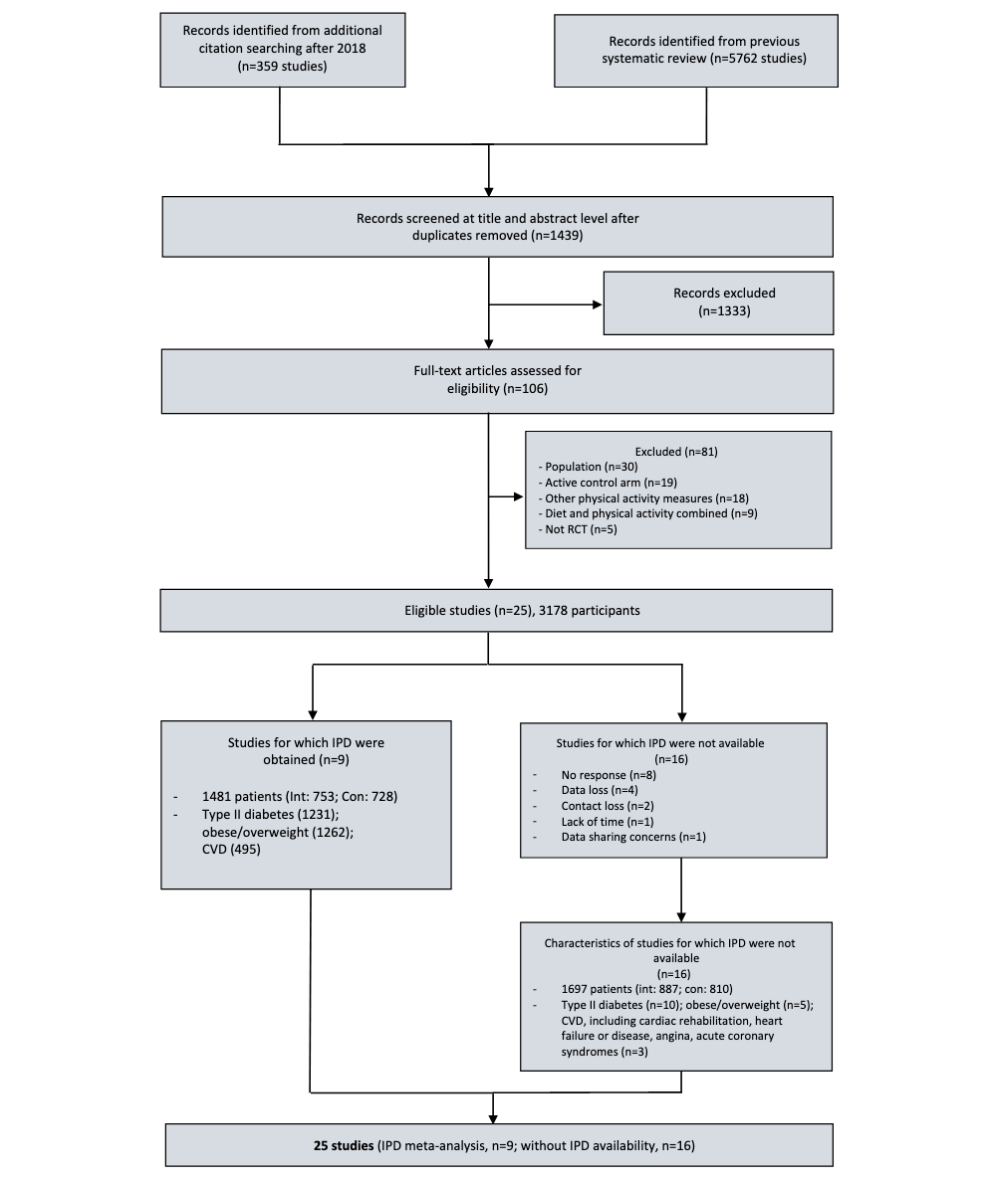
Identification and selection of studies providing individual participant data for meta-analysis of interventions involving wearable trackers for measuring steps per day in patients with cardiometabolic conditions. Con: control group; CVD: cardiovascular disease; int: intervention group; IPD: individual participant data; RCT: randomized controlled trial.

Of 25 eligible RCTs (including 3178 participants) that used wearable trackers and measured steps per day, we found that 9 studies (36%) had a median intervention duration of 12 (range 7-52) weeks, providing IPD for 47% of the total participants (1481/3178) (Figure 1). A list of the eligible studies is provided in Multimedia Appendix 1 (pages 12-14).

### Characteristics of Studies

Of the 9 included IPD studies, 5 were from North America [35-40], 3 were from the United Kingdom [41-43], and 1 was from Nigeria [44]. Wearable trackers were used in all 9 studies [35,37-44] and the primary outcome was steps per day. Within the IPD sample, 822 of 1481 patients were men (56%) and 907 of 1481 were 60 years or older (61%; range 25-86 years). A total of 1231 of 1481 patients had (or were at risk of) type 2 diabetes (83%), 1262 of 1481 were obese or overweight (85%), and 495 of 1481 patients had CVD (33%). Characteristics of the studies that provided IPD and did not provide IPD are presented in Multimedia Appendix 1 (pages 15-22); baseline characteristics of the IPD are provided in Table 1.

**Table 1:** Baseline characteristics of the individual participant data and imbalance assessment between treatment arms. Percentages are proportions of observations to intervention or control arms, as applicable.

Characteristics			Intervention		Control		*P* value^a^	
Steps per day (in 1481 patients in 9 studies) (n), mean (SD)			6071.25 (3060.72)		6072.11 (3064.40)		.99	
Age (in 1481 patients in 9 studies) (years), mean (SD)			60.53 (9.70)		60.73 (10.06)		.68	
Height (in 986 patients in 5 studies) (cm), mean (SD)			124.00 (74.12)		126.73 (72.35)		.56	
BMI (in 1325 patients in 7 studies) (kg/cm^2^), mean (SD)			32.03 (5.44)		32.11 (5.03)		.77	
**BMI by classification (in 1325 patients in 7 studies)**								.40
	Patients, N	680		645				
	Normal (18.5-24.9 kg/m^2^), n (%)	37 (5.4)		26 (4)				
	Overweight (25-29.9 kg/m^2^), n (%)	266 (39.1)		267 (41.4)				
	Obese (≥30 kg/m^2^), n (%)	377 (55.4)		352 (54.6)				
**Ethnicity (in 1414 patients in 9 studies)**								.27
	Patients, N	721		693				
	White European or North American, n (%)	534 (74.1)		510 (73.6)				
	African American, n (%)	105 (14.6)		116 (16.7)				
	Hispanic or Latino, n (%)	40 (5.5)		23 (3.3)				
	Mixed ethnicity, n (%)	27 (3.7)		29 (4.2)				
	Asian/Middle Eastern, n (%)	15 (2.1)		15 (2.2)				
**Education status^a^ (in 407 patients in 2 studies)**								.60
	Patients, N	210		183				
	Low (not completed secondary education to A level), n (%)	42 (20)		33 (18)				
	Medium (completed secondary education; ie, A level equivalent), n (%)	36 (17.1)		36 (19.7)				
	High (any further or higher education), n (%)	94 (45.8)		71 (38.8)				
Preexisting CVD (in 495 patients in 3 studies), n/N (%)			44/230 (19.1)		84/265 (32)		.07	
Preexisting type 2 diabetes (in 1231 patients in 4 studies), n/N (%)			370/619 (59.8)		403/612 (65.8)		.21	
Preexisting hypertension (in 642 patients in 3 studies), n/N (%)			250/311 (80.4)		264/331 (79.8)		.83	
Preexisting metabolic syndrome (in 471 patients in 2 studies), n/N (%)			194/240 (80.8)		201/231 (87)		.73	
Depression score (in 347 patients in 2 studies), mean (SD)			2.24 (4.13)		2.29 (4.10)		.99	
Smokers (in 578 patients in 3 studies), n/N (%)			87/282 (30.9)		84/296 (28.4)		.48	

^a^Mean values were compared with a 2-tailed *t* test and categorical covariates were compared with the chi-squared test or ANOVA.

### Risk of Bias Assessments

The RoB assessment of studies that contributed IPD compared with those that did not showed that the former had lower RoB across all domains (Multimedia Appendix 1, page 22). The assessments for each of the RoB domains of the IPD studies are available in Multimedia Appendix 1 (page 23), and the results of RoB assessments for non-IPD studies are available in our earlier systematic review [19].

### Efficacy of Wearable Trackers on Increasing Steps per Day

**Figure 2:**
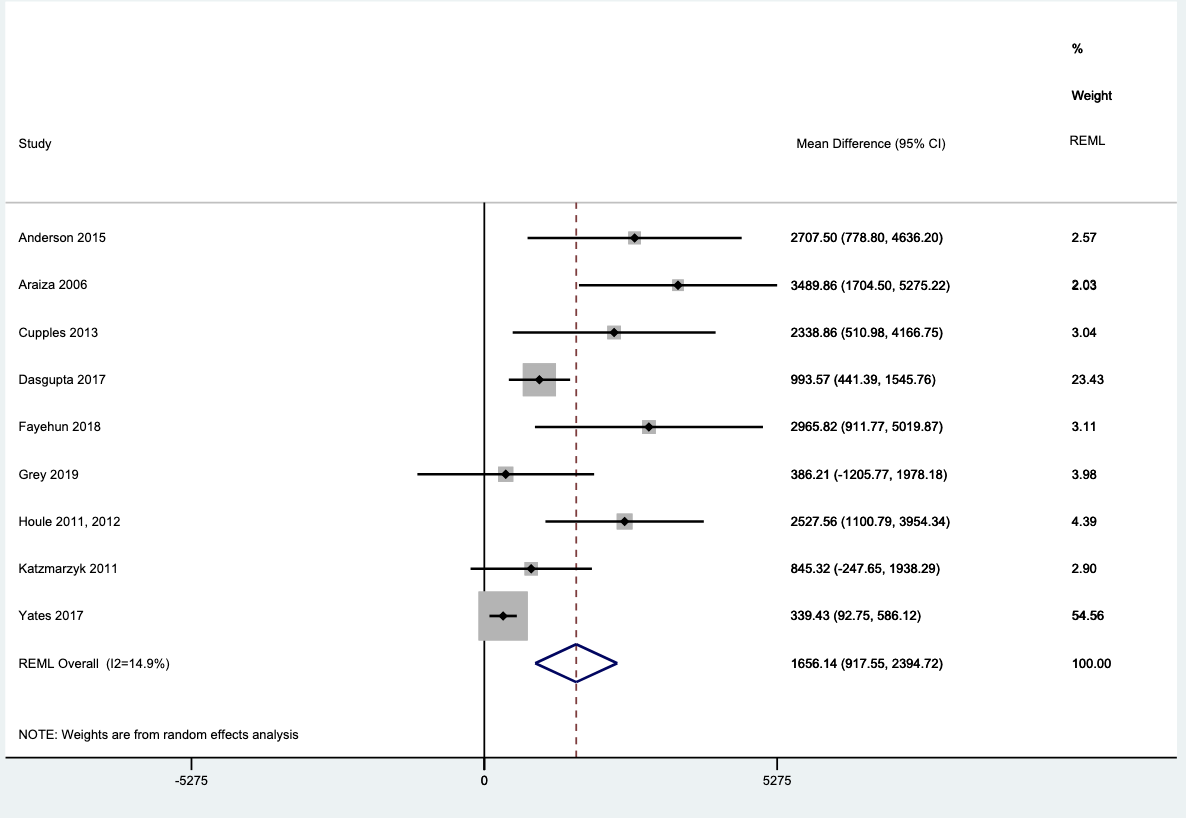
Forest plot showing 1-stage meta-analysis of individual participant data from studies using wearable trackers to measure steps per day; the mean postintervention difference in steps per day is also shown. MD: mean difference; REML: restricted maximum likelihood.

In the 1-stage analysis involving all 9 studies and 1481 participants, wearable trackers were associated with small but significantly improved levels of physical activity over the median intervention duration of 12 (range 6-52) weeks. The number of steps was 1656 (95% CI 918-2395) greater in the intervention than the control group (Figure 2). These results are consistent with the 2-stage analysis (Multimedia Appendix 1, page 32). The mean number of steps per day at the end of treatment, without adjusting for baseline scores, was 6561 (SD 3336) in the wearable activity tracker intervention group and 6561 (SD 3340) in the control group; this was not a significant difference (*P*=.99). Visual and statistical evidence (*P*=.002; Egger test) of a small study effect was found in the funnel plots (Multimedia Appendix 1, pages 24-30). After removing the studies with high or unclear RoB from the analysis, the symmetry of the funnel plot improved somewhat (*P*=.06). Secondary outcomes showed insignificant differences (Multimedia Appendix 1, page 31).

Covariate interaction effects for the primary outcome showed that patients older than 50 years benefited more from using wearable trackers. The interaction coefficient for patients aged 50 to 59 years was 1175.16 (95% CI 377.46 to 1972.86) steps per day, with an individual group effect of 2006.83 (95% CI 1163.83 to 2849.82); in patients aged 60 to 69 years, the interaction coefficient was 981.37 (95% CI 222.39 to 1740.35), with an individual group effect of 1813.04 (95% CI 986.40 to 2639.68); and in patients aged 70 to 90 years, the interaction coefficient was 1059.98 (95% CI 200.29 to 1919.66), with an individual group effect of 1891.65 (95% CI 963.98 to 2819.31; *I*^2^ 15.5%). In contrast, for patients aged under 50 years the interaction coefficient was 831.67 (95% CI –97.00 to 1760.33) (Table 2).

**Figure 3:**
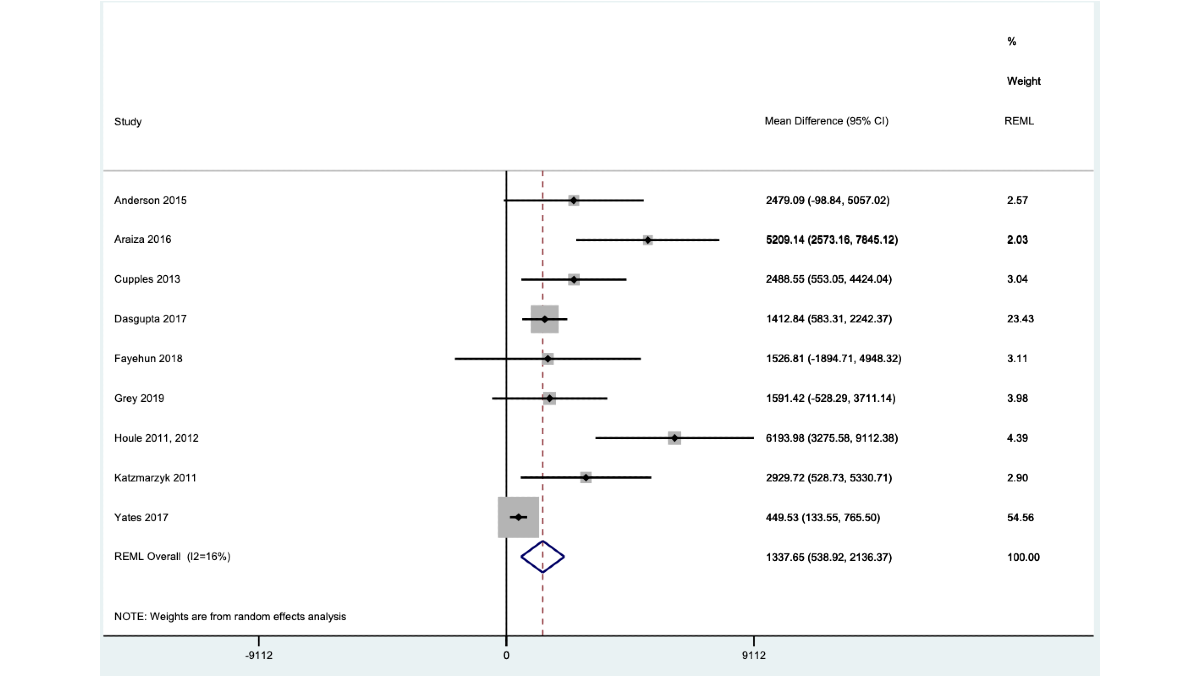
Gender effect for women. Forest plot showing 1-stage meta-analysis of individual participant data from women only, derived from studies using wearable trackers to measure steps per day; the mean postintervention difference in steps per day is also shown. MD: mean difference; REML: restricted maximum likelihood.

**Figure 4:**
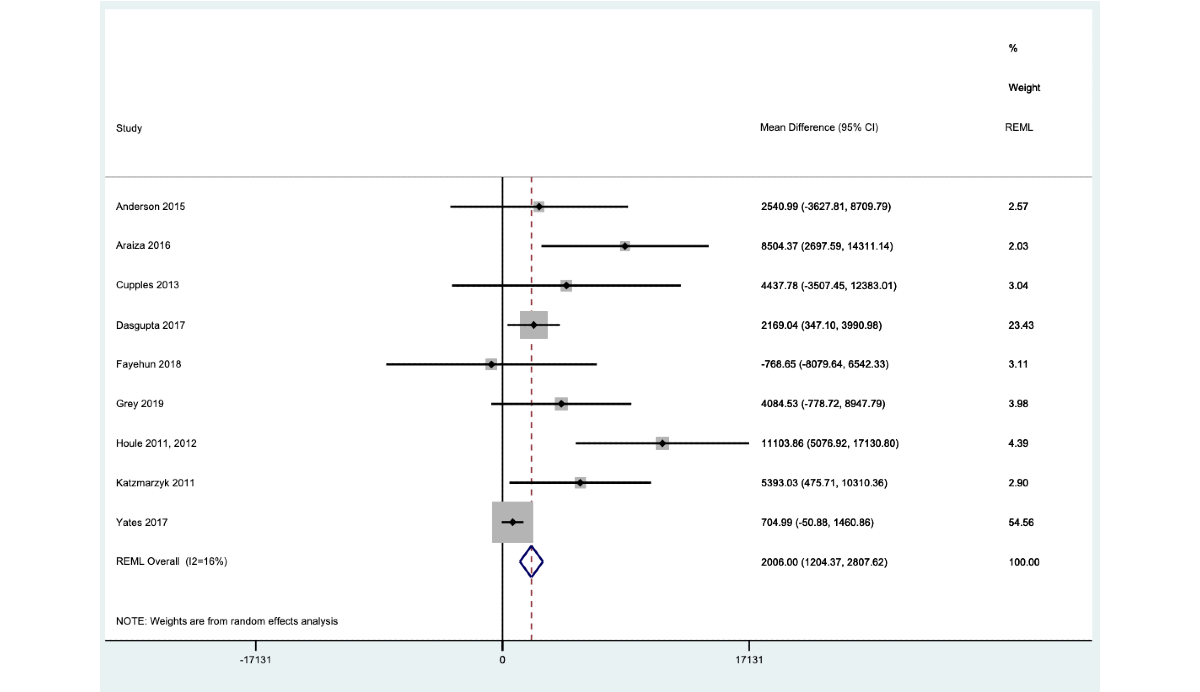
Gender effect for men. Forest plot showing 1-stage meta-analysis of individual participant data from men only, derived from studies using wearable trackers to measure steps per day; the mean postintervention difference in steps per day is also shown. MD: mean difference; REML: restricted maximum likelihood.

The number of steps per day after using wearable trackers was greater among men than women (interaction coefficient –668.3, 95% CI –1156.8 to –179.93). For men, the mean number of steps per day was 2006 (95% CI 1204.4 to 2807.6), while for women, it was 1337.65 (95% CI 538.92 to 2136.37; *I*^2^ 16%) (Figure 3 and Figure 4). Patients with at least 2 comorbidities showed a significantly lower number of steps per day; the interaction coefficient for 2 comorbidities was –516.80 (95% CI –1188.34 to –10.74), and the mean number of steps was 1344.70 (95% CI 421.62 to 1843.87). For patients with 3 comorbidities, the interaction coefficient was –876.44 (95% CI –2071.88 to 509.41), and the mean number of steps was 570.17 (95% CI –304.66 to 870.08; *I*^2^ 15.7%). In contrast, for patients with only 1 comorbidity, the interaction coefficient was 1861.55 (95% CI 1061.6 to 2661.5). White patients also displayed a higher step count after using wearable trackers (2189, 95% CI 1276 to 3102) compared to other ethnic groups (1194, 95% CI 280 to 2107); the interaction coefficient was 995 (95% CI 360 to 1631) steps per day (*I*^2^ 21%).

**Table 2:** Differential effects of wearable trackers on physical activity measured by steps per day among specific subgroups of patients.

Characteristic			Mean difference in steps per day,^a^ n (95% CI)		Treatment covariate interaction				
					Interaction coefficient (95% CI)		*P* value		*I*^2^, % (95% CI)
**Age**									
	≥60 years	1814.39 (996.51 to 2632.28)		1		N/A^b^		16.1 (5.0 to 41.1)	
	<60 years^c^	1566.83 (766.98 to 2366.68)		–247.56 (–762.0 to 266.92)		.35			
**Age category**									
	20-49 years	831.67 (–97.00 to 1760.33)		1		N/A		15.5 (4.8 to 40.2)	
	50-59 years	2006.83 (1163.83 to 2849.82)		1175.16 (377.46 to 1972.86)		.004			
	60-69 years	1813.04 (986.40 to 2639.68)		981.37 (222.39 to 1740.35)		.01			
	70-90 years	1891.65 (963.98 to 2819.31)		1059.98 (200.29 to 1919.66)		.02			
**Sex**									
	Men	2006 (1204.4 to 2807.6)		1		N/A		16.03 (5.0 to 40.91)	
	Women	1337.65 (538.92 to 2136.37)		–668.3 (–1156.8 to –179.93)		.01			
**Ethnicity**									
	Other	1193.65 (280.31 to 2106.99)		1		N/A		20.5 (7.0 to 46.8)	
	White^d^	2189.0 (1276.3 to 3101.65)		995.30 (359.80 to 1630.80)		.002			
**Comorbidities^e^**									
	1	1861.55 (1061.6 to 2661.5)		1		N/A		15.7 (4.6 to 41.7)	
	2	1344.70 (421.62 to 1843.87)		–516.80 (–1188.34 to –10.74)		.04			
	3	570.17 (–304.66 to 870.08)		–876.44 (–2071.88 to –509.41)		.01			
	4	1078.28 (468.72 to 2077.31)		N/A		N/A			
**Cardiometabolic condition focus**									
	Other conditions^f^	1535.28 (–557.35 to 3627.91)		1		N/A		16.2 (0.5 to 87.3)	
	Type II diabetes	1942.47 (47.24 to 3837.70)		407.19 (–785.09 to 1599.47)		.50			

^a^Model accounted for baseline steps per day scores with analysis of covariance.

^b^N/A: not applicable.

^c^Per year of age.

^d^White versus all other ethnicities.

^e^Including type II diabetes, hypertension, angina, obese or overweight, and any other cardiovascular condition (excluding stroke).

^f^Including hypertension.

### Further Sensitivity Analysis and Exploratory Analysis

Behavior change frameworks were used in 3 studies [35,37,38,45], but did not appear to improve the number of steps per day (the interaction coefficient was –1054, 95% CI –2785 to 677). Nevertheless, groups that did or did not use the program still showed a statistically significant change (part of program: 2476, 95% CI 935 to 4017; not part of program: 1422, 95% CI 634 to 2211). In addition, studies that set goals for reaching specific steps per day also did not appear to improve the number of steps per day, nor did the placement of the wearable tracker (ie, waist vs wrist) (Multimedia Appendix 1, page 33).

Small but nonsignificant improvements in steps per day were seen in studies with low RoB compared to studies with high or unclear RoB. Studies that assessed the number of steps per day beyond 26 weeks showed a lower (but nonsignificant) number of steps per day compared to studies that assessed performance in the short term (ie, less than 6 months). While both groups showed statistically significant changes (<26 weeks: 2000, 95% CI 1068 to 2932; ≥26 weeks: 1143, 95% CI 33 to 2254) when they used trackers over the longer term (ie, beyond 6 months) it was clear that performance waned by almost 1000 steps per day compared to short-term use (ie, up to 6 months). Studies that involved face-to-face delivery of the wearable tracker significantly improved the number of steps per day (2630, 95% CI 1835 to 3425) when compared to studies that involved only self-regulated use of the tracker (850, 95% CI 325 to 1375) (the interaction coefficient was 1780, 95% CI 826 to 2733).

Improvement in steps per day among the non-IPD studies was significantly higher with the wearable trackers; results showed almost double the number of steps per day (2854, 95% CI 1944 to 3763) compared to the IPD meta-analysis estimate (Multimedia Appendix 1, page 34). However, including the non-IPD studies in the meta-analysis increased bias from small studies and worsened the funnel plot asymmetry (Multimedia Appendix 1, pages 26-27).

## Discussion

### Principal Findings

This IPD meta-analysis confirms that interventions using wearable trackers were effective at increasing physical activity, as measured by total number of steps per day, in participants with cardiometabolic conditions, compared to control groups, over a median duration of 12 weeks. These improvements were slightly lower than estimated by our aggregate-data meta-analysis [19]. In this IPD meta-analysis, we identified differential effects in relation to age, sex, and ethnicity of the participants and the number of comorbidities present. Consistent with our aggregate-data meta-analysis [19], we observed that interventions that used wearable trackers with face-to-face delivery by a professional were more effective at increasing steps per day than patient self-managed interventions.

The benefits in terms of the mean difference in steps per day after using wearable trackers in the short term were not as large as seen in other meta-analyses [46,47], but at the end of treatment, the activity tracker mean score increased to 6561 steps per day, which does appear to meet the recommended number of daily steps as outlined in public health guidelines [48,49]. For example, the average daily steps recorded were just above the recommendation of 3000 to 6000 steps per day made by the United Kingdom’s National Obesity Forum and the recommendation of 3000 steps per day made by Northern Ireland’s Public Health Agency [50]. However, the large standard deviation (3336) from this mean score suggests that some patients were still underachieving and did not meet the goal for steps per day. Still, recent evidence [51] has shown that for each daily increase of 1000 steps in physically inactive individuals at baseline, the estimated risk reduction in all-cause mortality is 6% to 36%, while the reduction for CVD is 5% to 21%. An increase of 500 steps per day or the equivalent (eg, 5 minutes of brisk walking) is also considered the minimum clinically important difference in steps per day in inactive adults [52]. Further, a recent dose-response meta-analysis [53] of the association between steps per day and all-cause mortality risk indicated a strong inverse association; the risk decreased linearly from 2700 to 17,000 steps per day. More specifically, the hazard ratio for 10,000 steps per day was 0.44 (95% CI 0.31 to 0.63). Even the most extremely physically inactive patients, such as the ones in our study, are still likely to benefit from small gains in steps per day.

Wearable trackers were predominately more effective in White men, but were still somewhat effective in non-White men and women. Evidence gathered from various countries shows that women are less active than men (there is a global average of 31.7% inactive women vs 23.4% inactive men) and that barriers to women’s involvement in sports are numerous and complex [54-56]. Men tend to have more intrinsic motivators linked to and leading to physical activity, such as the need or desire to improve health, prevent disease onset, and improve body shape, and are also more competitive [57]. In contrast, different stimuli appear to motivate women of various ages to undertake physical exercise, such as emotional involvement, socialization, mental and physical well-being, and the achievement and maintenance of a positive self-image [58]. Women may also have less time due to daily household chores. Therefore, policies that address the sex gap in physical activity could start with better access and investment and by altering sociocultural norms. In relation to ethnicity, there is evidence that non-White participants have lower levels of physical activity and that their participation in and benefit from physical activity programs are suboptimal, due to lower access and socioeconomic and sociocultural bias [59]. Our findings suggest that non-White women in particular are less likely to achieve significant benefits from interventions involving wearable trackers. This may be because of the increased likelihood that they encounter social, economic, and cultural barriers to physical activity that are unique to them.

Wearable trackers were also most effective for improving physical activity in participants aged 49 years or more, and were surprisingly ineffective in the 365 participants aged 50 years or less. Although this could be a sample size issue, there are some possible explanations for this finding. People under 50 years old may be less likely to engage with wearable trackers due to caring and work responsibilities, and they may have less time to participate in daily physical activity [60]. Conversely, patients aged between 50 and 60 years generally maintained better daily physical activity levels than those over the age of 60. This may be due to multiple comorbidities, which are likely to be the leading cause of reduced physical activity in the older age group [61].

Participants with a cardiometabolic condition have a relatively higher likelihood of developing additional comorbidities, meaning that many of these high-risk patients are often diagnosed with 2 or more diseases [62]. As we have clearly shown in this study, most patients do not meet the international clinical guidelines for recommended steps per day, and when cardiometabolic risk is combined with a comorbidity, the steps-per-day performance was reduced even further. This result reinforces several key studies and policy guidelines, which show that a worsening steps-per-day performance is highly associated with multimorbidity [1,2,5,6,63]. Moreover, it is well known that low physical activity increases weight, BMI, and waist circumference, which are all key predictors for further exacerbation of comorbid chronic diseases, including diabetes, hypertension, and dyslipidemia [64]. For health care professionals, effective and practical management of patients with multimorbidity is important. A more sensitive understanding of their lifestyles and their tendency toward extremely low levels of physical activity will facilitate the support of those most in need of it.

Secondary cardiometabolic biomarkers, such as blood sugar glucose, measured as hemoglobin A_1c_ (HbA_1c_), blood pressure, cholesterol, and BMI or weight, were all found to not be statistically significant, which may be unsurprising given the short duration of the interventions (only 12 weeks). However, one study [65] did find small but significant reductions in BMI and systolic blood pressure when using pedometers, although the patient population in this study involved a variety of outpatients, who may not have been suffering from the same severe underlying cardiometabolic health conditions. Another study [47] investigated the effectiveness of setting physical activity goals in patients with type 2 diabetes who used step counters and did not report any significant reduction in HbA_1c_ level. Similarly, a meta-analysis [46] that compared accelerometers and pedometers for improving physical activity levels and HbA_1c_ levels in people with type 2 diabetes showed no significant differences between either type of tracker. While our original review [19] did show significant reductions in HbA_1c_ levels, this can only be considered a small reduction in effect size, and is not dissimilar to our findings based on IPD (–0.19 vs –0.13).

### Strengths and Limitations

This is the first IPD meta-analysis to assess the differential effects of wearable trackers for important physical activity and clinically relevant outcomes in participants with cardiometabolic conditions. Strengths include a clear standardization of definitions and outcomes, imputation of missing data, a robust analysis that included ANCOVA [66], exploration of the potential for differential effects [29], extensive data checking and work with study investigators to ensure the quality of the data set, and the inclusion of studies that mostly had low RoB. Nevertheless, we were unable to obtain IPD from 16 studies, which meant that 1697 of 3178 (53%) of potential patients were missing. However, these studies were mainly small and generally had a higher RoB than the included studies. Over two-thirds of the IPD were from White participants, meaning that all other ethnic groups (Black African, Asian, Hispanic, and others) had to be combined into one category to allow for adequate power in the subgroup analysis. Nevertheless, we found that White patients had significantly higher step counts than other ethnic groups. This result is similar to findings from a recent prospective cohort study that assessed the association of steps per day with premature all-cause mortality among Black and White men and women with coronary artery risk and showed that Black participants took fewer steps than White participants (median 8670 steps/day, IQR 6810 to 10,811, vs median 9441 steps/day, IQR 7704 to 11,329, respectively; *P*<.001) [67]. As 83% of the patients had or were at risk of type 2 diabetes and 85% of the patients were at least overweight or obese, this meant that these 2 conditions largely overlapped in the patient population, meaning that it was not possible to properly adjust for this in the analysis. In addition, as only 33% of the patients had CVD, and no other condition data were provided in the IPD, these 2 conditions could only be compared with CVD. Thus, we urge that these results are interpreted with some caution and encourage better coding of condition data, which would allow for more detailed analyses. Only 3 studies [37,38,42,43] used a behavior change framework as part of their intervention design, and only 3 studies [36-38,43] collected data over the longer term (ie, at least 1 year). Both are clear weaknesses that limit our understanding of sustained effects over time, which is an important gap in knowledge [68]. Differences in tracker functionality may also have significant effects on their performance. For instance, trackers are often criticized for not measuring daily steps precisely enough, particularly when the tracker becomes tilted below the waistline in overweight or obese individuals [69-71]. However, some more expensive wearable trackers that can sense movement in a tilted position have shown promise [72]. Diversity in wearing time and self-monitoring across the studies made it impossible to effectively categorize this information for meaningful subgroup analyses.

Moreover, following an update to our aggregate review, we found that 8 studies, which included 414 participants, used wearable trackers to measure physical activity with variable measurement outputs rather than steps per day. We excluded these 8 studies from our IPD meta-analysis because the assessment of the differential effects would have been underpowered, with only 414 participants available. Once the evidence base is more developed, we strongly encourage future efforts to compare our findings with those of studies using additional measurement outputs or other wearable technologies (eg, smartphone apps, smartwatches, and wristbands). Finally, our last search update (in December 2020) could be considered marginally outdated for an aggregate-data meta-analysis. However, data acquisition, preparation, and analysis for IPD meta-analyses requires considerably more time and resources than aggregate-data meta-analyses. We strongly recommend universal open access to trial data to speed access to IPD in the future.

### Conclusion

Interventions using wearable trackers were effective at providing a small mean improvement in steps per day over short periods of use in participants with a cardiometabolic condition. Interventions with wearable trackers that were delivered and guided by a professional were most effective in White men and in those aged 50 years and older with only one comorbidity. Future research should look at ways to extend the beneficial effects of interventions with wearable trackers to other patients (particularly women) with cardiometabolic conditions and for longer periods.

## Appendix

Multimedia Appendix 1 Supplementary appendix booklet.

## Abbreviations

ANCOVA: analysis of covariance
CVD: cardiovascular disease
HbA_1c_: hemoglobin A_1c_
IPD: individual participant data
MD: mean difference
RCT: randomized controlled trial
RoB: risk of bias

## Data Availability

A data sharing agreement form was completed by each participant that provided individual participant data; there has not been any agreement to share the data. Therefore, authors seeking individual participant data should contact the principal investigators from the individual trials if they desire access. However, we aim to discuss with the authors the possibility of making the individual participant data database publicly available.

## Disclaimer

This paper presents independent research funded by the National Institute for Health Research School for Primary Care Research Project 390. The views expressed are those of the authors and not necessarily those of the National Institute for Health Research or the Department of Health and Social Care. The research team members were independent from the funding agencies. The funders had no role in the design and conduct of the study; the collection, management, analysis, and interpretation of the data; or the preparation, review, and approval of the manuscript.
