# Supplementary material for: Association Between Patient Factors and the Effectiveness of Wearable Trackers at Increasing the Number of Steps per Day Among Adults With Cardiometabolic Conditions: Meta-analysis of Individual Patient Data From Randomized Controlled Trials
Source: J Med Internet Res. 2022 Aug 30;24(8):e36337. doi: 10.2196/36337 (PMC9472038; doi:10.2196/36337)
Supplement: Multimedia Appendix 1 [file jmir_v24i8e36337_app1.docx]

**Supplementary appendix**

Contents

[Appendix 1: Medline search strategy 3](#_Toc95209213)

[Appendix 2: Citations of included IPD and non-IPD studies 12](#_Toc95209214)

[Appendix 3: Characteristics of eligible randomised trials 15](#_Toc95209215)

[Appendix 4: Assessment of risk of bias 22](#_Toc95209216)

[Appendix 5: Assessment of publication bias 24](#_Toc95209217)

[Appendix 6: Secondary outcome IPD-MAs results 31](#_Toc95209218)

[Appendix 7: Two stage IPD meta-analysis 32](#_Toc95209219)

[Appendix 8: Intervention and programme subgroup factors and other sensitivity analysis 33](#_Toc95209220)

[Appendix 9: Studies not contributing IPD 34](#_Toc95209221)

[Appendix 10: List of collaborators 35](#_Toc95209222)

[Appendix 11: IPD PRISMA checklist 39](#_Toc95209223)

# Appendix 1: Medline search strategy

**Medline**

| **# ▲** | **Searches** | **Results** |
| --- | --- | --- |
| 1 | Cardiovascular Diseases/ | 152632 |
| 2 | (cardiovascular adj1 disease$).tw. | 150229 |
| 3 | cardiovascular risk factor$.tw. | 31197 |
| 4 | exp heart diseases/ | 1146729 |
| 5 | exp Coronary Artery Bypass/ | 53473 |
| 6 | exp Myocardial Revascularization/ | 92869 |
| 7 | exp heart transplantation/ | 36070 |
| 8 | Percutaneous Coronary Intervention/ or Angioplasty, Balloon, Coronary/ | 54555 |
| 9 | Heart Valve Prosthesis/ | 36394 |
| 10 | Pulmonary embolism/ | 39358 |
| 11 | ((myocardial or cardiac or heart) adj2 (infarct* or isch?emi*)).tw. | 239817 |
| 12 | (coronary adj2 (syndrome* or disease* or event* or occlusion* or stenos* or thrombo*)).tw. | 178348 |
| 13 | (myocard* adj2 revasculari?ation).tw. | 5352 |
| 14 | (STEMI or NSTEMI).tw. | 10192 |
| 15 | (ST adj2 (elevat* or depress*)).tw. | 29827 |
| 16 | "heart transplant*".tw. | 21509 |
| 17 | angina.tw. | 49402 |
| 18 | (heart adj2 (failure or attack or bypass or disease*)).tw. | 294547 |
| 19 | ((heart or cardiac or myocard*) adj2 (fail* or insufficien* or decomp*)).tw. | 168633 |
| 20 | (HFpEF or HFrEF or left ventricular ejection fraction or ((preserved or reduced) adj ejection fraction)).tw. | 28582 |
| 21 | (LV dysfunction or (diastolic adj (dysfunction* or failure*)) or (systolic adj (dysfunction* or failure*))).tw. | 17690 |
| 22 | pulmonary embolism*.tw. | 29722 |
| 23 | CABG.tw. | 16083 |
| 24 | (coronary adj2 bypass).tw. | 43973 |
| 25 | PTCA.tw. | 6211 |
| 26 | angioplast*.tw. | 40523 |
| 27 | PCI.tw. | 22324 |
| 28 | (Percutaneous adj2 intervention*).tw. | 32511 |
| 29 | (stent* adj3 (heart or cardiac*)).tw. | 805 |
| 30 | (heart valve adj1 (device* or artificial or prosthesis)).tw. | 667 |
| 31 | cardiomyopath*.tw. | 63613 |
| 32 | cardiovascular disease*.tw. | 149898 |
| 33 | or/1-32 | 1570796 |
| 34 | Diabetes mellitus/ | 119569 |
| 35 | diabet*.ti. | 306993 |
| 36 | exp Diabetes Mellitus, Type 2/ | 137087 |
| 37 | ((type 2 or type ii) adj2 diabet*).ti,ab. | 124078 |
| 38 | ((non insulin* depend* or non insulin* depend* or non-insulin?depend* or non insulin?depend*) adj1 diabet*).ti,ab. | 9807 |
| 39 | (T2DM or T2D or TIIDM or TIID or NIDDM or MODY or MODM or AODM).ti,ab. | 31996 |
| 40 | ((obes* or overweight) adj5 diabet*).ti,ab. | 39051 |
| 41 | prediabetic state/ | 7067 |
| 42 | (prediabetes or pre diabetes or raised glucose intolerance or impaired glucose level$ or impaired glucose tolerance or IGT or impaired fasting glucose or IFT or FPG or fasting plasma glucose or impaired glucose regulation or impaired glucose metabolism or raised glycated haemoglobin or raised glycated hemoglobin or high glycated Hb or hyperglycaemia or hyperglycemia).tw. | 75450 |
| 43 | ((prevent* or avoid* or delay* or decreas* or reduc*) adj2 (type II diabetes or type 2 diabetes or T2D or DM or diabetes)).ti,ab. | 12238 |
| 44 | or/34-43 | 463396 |
| 45 | exp Obesity/ | 218805 |
| 46 | Obese.tw. | 112489 |
| 47 | exp Overweight/ | 225397 |
| 48 | (BMI or body mass index).af. | 255484 |
| 49 | Weight gain/ | 31929 |
| 50 | (Overweight or over weight or obesity or adipose).af. | 416529 |
| 51 | exp Obesity/pc | 19636 |
| 52 | (body mass index or BMI).mp. | 254344 |
| 53 | or/45-52 | 595592 |
| 54 | Randomized Controlled Trial/ | 520611 |
| 55 | Clinical Trial/ | 526309 |
| 56 | randomized controlled trial.pt. or randomised controlled trial.mp. [mp=title, abstract, original title, name of substance word, subject heading word, floating sub-heading word, keyword heading word, organism supplementary concept word, protocol supplementary concept word, rare disease supplementary concept word, unique identifier, synonyms] | 525725 |
| 57 | controlled clinical trial.pt. | 93998 |
| 58 | trial*.ti,ab. | 916198 |
| 59 | or/54-58 | 1474132 |
| 60 | pedomet*.mp. | 2396 |
| 61 | ((step* or walk*) adj2 (count* or sensor or meter)).ti,ab. | 2850 |
| 62 | Accelerometry/ or (accelerom* or actimeter or actigraph or actiwatch or GT3X or fitbit).ti,ab. | 16461 |
| 63 | ((activit* or move* or motion or energy or exercise) adj2 (monitor* or sens* or detect* or count*)).tw. | 63488 |
| 64 | or/60-63 | 81215 |
| 65 | 33 or 44 or 53 | 2390948 |
| 66 | 59 and 64 and 65 | 2120 |
| **67** | **limit 66 to yr="2000 - current"** | **1926** |

**Embase**

| **# ▲** | **Searches** | **Results** |
| --- | --- | --- |
| 1 | Diabetes mellitus/ | 548816 |
| 2 | diabet*.ti. | 459899 |
| 3 | exp Diabetes Mellitus, Type 2/ | 267837 |
| 4 | ((type 2 or type ii) adj2 diabet*).ti,ab. | 235422 |
| 5 | ((non insulin* depend* or non insulin* depend* or non-insulin?depend* or non insulin?depend*) adj1 diabet*).ti,ab. | 11649 |
| 6 | (T2DM or T2D or TIIDM or TIID or NIDDM or MODY or MODM or AODM).ti,ab. | 70722 |
| 7 | ((obes* or overweight) adj5 diabet*).ti,ab. | 70600 |
| 8 | prediabetic state/ | 14073 |
| 9 | (prediabetes or pre diabetes or pre-dm or subclinical diabetic or raised glucose intolerance or impaired glucose level$ or impaired glucose tolerance or IGT or impaired fasting glucose or IFT or FPG or fasting plasma glucose or impaired glucose regulation or impaired glucose metabolism or raised glycated haemoglobin or raised glycated hemoglobin or high glycated Hb or hyperglycaemia or hyperglycemia or without diabet* or without diagnosed diabet*).tw. | 145300 |
| 10 | ((prevent* or avoid* or delay* or decreas* or reduc*) adj2 (type II diabetes or type 2 diabetes or T2D or DM or diabetes)).ti,ab. | 20813 |
| 11 | or/1-10 | 1008275 |
| 12 | exp Obesity/ | 532605 |
| 13 | Obese.tw. | 201114 |
| 14 | (overweight or obese or over-weight or over weight or overeating or over eating or over-eating).ti. | 75901 |
| 15 | exp Overweight/ | 532605 |
| 16 | (BMI or body mass index).af. | 474490 |
| 17 | exp weight reduction programs/ | 2431 |
| 18 | Weight gain/ | 87925 |
| 19 | (Overweight or over weight or obesity or adipose).af. | 683883 |
| 20 | exp Obesity/pc | 16147 |
| 21 | (body mass index or BMI).mp. | 470733 |
| 22 | Cardiovascular Diseases/ | 31817 |
| 23 | (cardiovascular or cv or cvd or vascular or coronary).tw. | 1824792 |
| 24 | heart disease$.tw. | 225534 |
| 25 | cardiovascular risk factor$.tw. | 56404 |
| 26 | or/12-19 | 1083238 |
| 27 | or/22-25 | 1941192 |
| 28 | Randomized Controlled Trial/ | 638226 |
| 29 | Clinical Trial/ | 988522 |
| 30 | randomized controlled trial.pt. or randomised controlled trial.mp. [mp=title, abstract, heading word, drug trade name, original title, device manufacturer, drug manufacturer, device trade name, keyword, floating subheading word, candidate term word] | 34253 |
| 31 | controlled clinical trial/ | 466002 |
| 32 | trial*.ti,ab. | 1517240 |
| 33 | pedomet*.mp. | 4327 |
| 34 | ((step* or walk*) adj2 (count* or sensor or meter)).ti,ab. | 5971 |
| 35 | Accelerometry/ or (accelerom* or actimeter or actigraph or actiwatch or GT3X or fitbit).ti,ab. | 27313 |
| 36 | ((activit* or move* or motion or energy or exercise) adj2 (monitor* or sens* or detect* or count*)).tw. | 87436 |
| 37 | 11 or 26 or 27 | 3397736 |
| 38 | or/28-32 | 2324948 |
| 39 | or/33-36 | 118012 |
| 40 | 37 and 38 and 39 | 2234 |
| **41** | **limit 40 to (year="2000 - current")** | **2081** |

**PsycInfo**

| **# ▲** | **Searches** | **Results** |
| --- | --- | --- |
| 1 | Diabetes mellitus/ | 5321 |
| 2 | diabet*.ti. | 14551 |
| 3 | exp Diabetes Mellitus/ | 8726 |
| 4 | ((type 2 or type ii) adj2 diabet*).ti,ab. | 8188 |
| 5 | ((non insulin* depend* or non insulin* depend* or non-insulin?depend* or non insulin?depend*) adj1 diabet*).ti,ab. | 189 |
| 6 | (T2DM or T2D or TIIDM or TIID or NIDDM or MODY or MODM or AODM).ti,ab. | 1650 |
| 7 | ((obes* or overweight) adj5 diabet*).ti,ab. | 2742 |
| 8 | prediabetic state.tw. | 11 |
| 9 | (prediabetes or pre diabetes or pre-dm or subclinical diabetic or raised glucose intolerance or impaired glucose level$ or impaired glucose tolerance or IGT or impaired fasting glucose or IFT or FPG or fasting plasma glucose or impaired glucose regulation or impaired glucose metabolism or raised glycated haemoglobin or raised glycated hemoglobin or high glycated Hb or hyperglycaemia or hyperglycemia or without diabet* or without diagnosed diabet*).tw. | 4208 |
| 10 | ((prevent* or avoid* or delay* or decreas* or reduc*) adj2 (type II diabetes or type 2 diabetes or T2D or DM or diabetes)).ti,ab. | 1202 |
| 11 | or/1-10 | 22859 |
| 12 | exp Obesity/ | 25230 |
| 13 | Obese.tw. | 16441 |
| 14 | (overweight or obese or over-weight or over weight or overeating or over eating or over-eating).ti. | 7144 |
| 15 | exp Overweight/ | 26664 |
| 16 | (BMI or body mass index).af. | 62048 |
| 17 | Weight gain/ | 3210 |
| 18 | (Overweight or over weight or obesity or adipose).af. | 136755 |
| 19 | (body mass index or BMI).mp. | 33283 |
| 20 | exp Cardiovascular Disorders/ | 63480 |
| 21 | (cardiovascular or cv or cvd or vascular or coronary).tw. | 57571 |
| 22 | heart disease$.tw. | 10469 |
| 23 | cardiovascular risk factor$.tw. | 2354 |
| 24 | or/12-19 | 156833 |
| 25 | Randomized Controlled Trial.mp. | 19510 |
| 26 | randomized controlled trial.pt. or randomised controlled trial.mp. [mp=title, abstract, heading word, table of contents, key concepts, original title, tests & measures, mesh] | 3827 |
| 27 | trial*.ti,ab. | 187204 |
| 28 | pedomet*.mp. | 898 |
| 29 | ((step* or walk*) adj2 (count* or sensor or meter)).ti,ab. | 795 |
| 30 | Accelerometry/ or (accelerom* or actimeter or actigraph or actiwatch or GT3X).ti,ab. | 4253 |
| 31 | ((activit* or move* or motion or energy or exercise) adj2 (monitor* or sens* or detect* or count*)).tw. | 12367 |
| 32 | 11 or 24 | 170531 |
| 33 | 25 or 26 or 27 | 187468 |
| 34 | 28 or 29 or 30 or 31 | 17347 |
| 35 | 32 and 33 and 34 | 495 |
| **36** | **limit 35 to yr="2000 -Current"** | **492** |

**CENTRAL**

| **# ▲** | **Searches** | **Results** |
| --- | --- | --- |
| 1 | Diabetes mellitus/ | 9817 |
| 2 | diabet*.ti. | 58382 |
| 3 | exp Diabetes Mellitus/ | 31706 |
| 4 | ((type 2 or type ii) adj2 diabet*).ti,ab. | 38918 |
| 5 | ((non insulin* depend* or non insulin* depend* or non-insulin?depend* or non insulin?depend*) adj1 diabet*).ti,ab. | 1965 |
| 6 | (T2DM or T2D or TIIDM or TIID or NIDDM or MODY or MODM or AODM).ti,ab. | 10948 |
| 7 | ((obes* or overweight) adj5 diabet*).ti,ab. | 6269 |
| 8 | prediabetic state.tw. | 31 |
| 9 | (prediabetes or pre diabetes or pre-dm or subclinical diabetic or raised glucose intolerance or impaired glucose level$ or impaired glucose tolerance or IGT or impaired fasting glucose or IFT or FPG or fasting plasma glucose or impaired glucose regulation or impaired glucose metabolism or raised glycated haemoglobin or raised glycated hemoglobin or high glycated Hb or hyperglycaemia or hyperglycemia or without diabet* or without diagnosed diabet*).tw. | 17285 |
| 10 | ((prevent* or avoid* or delay* or decreas* or reduc*) adj2 (type II diabetes or type 2 diabetes or T2D or DM or diabetes)).ti,ab. | 4520 |
| 11 | or/1-10 | 80240 |
| 12 | exp Obesity/ | 14067 |
| 13 | Obese.tw. | 23680 |
| 14 | (overweight or obese or over-weight or over weight or overeating or over eating or over-eating).ti. | 14192 |
| 15 | exp Overweight/ | 15901 |
| 16 | (BMI or body mass index).af. | 64683 |
| 17 | Weight gain/ | 2496 |
| 18 | (Overweight or over weight or obesity or adipose).af. | 48579 |
| 19 | (body mass index or BMI).mp. | 64681 |
| 20 | (cardiovascular or cv or cvd or vascular or coronary).tw. | 139427 |
| 21 | heart disease$.tw. | 17801 |
| 22 | cardiovascular risk factor$.tw. | 5514 |
| 23 | or/12-22 | 95752 |
| 24 | pedomet*.mp. | 1861 |
| 25 | ((step* or walk*) adj2 (count* or sensor or meter)).ti,ab. | 1940 |
| 26 | Accelerometry/ or (accelerom* or actimeter or actigraph or actiwatch or GT3X or fitbit).ti,ab. | 5008 |
| 27 | ((activit* or move* or motion or energy or exercise) adj2 (monitor* or sens* or detect* or count*)).tw. | 7415 |
| 28 | 11 or 23 | 106036 |
| 29 | 24 or 25 or 26 or 27 | 9390 |
| 30 | 28 and 29 | 1210 |
| **31** | **limit 30 to yr="2000 - current"** | **1171** |

Bibliographic searches of CINAHL from 2000 until **December 2020** was also done.

Trial registers (ClinicalTrials.gov, ISCTRN, the WHO ICTRP portal and OpenTrial.net) were also searched to identify unpublished and/or ongoing trials. Authors of included trials were asked to identify any unpublished trials of which they were aware. References from recent relevant systematic reviews and aggregate data meta-analyses were checked for eligible trials.

# Appendix 2: Citations of included IPD and non-IPD studies

**References of studies with wearable tracker measuring step-per-day and providing IPD**

1. Anderson D.R. Health Beliefs, Will to Live, Hope, and Social Support in a Pedometer-Based Exercise Intervention among Cardiac Rehabilitation Patients. 2015. Available at: https://etd.ohiolink.edu/!etd.send_file?accession=osu1434901973&disposition=inline.
2. Araiza P, Hewes H, Gashetewa C, et al. Efficacy of a pedometer-based physical activity program on parameters of diabetes control in type 2 diabetes mellitus. *Metabolism* 2006;55(10):1382-7.
3. Cupples ME, Dean A, Tully MA, et al. A feasibility study of a randomized controlled trial of a pedometer based exercise intervention to promote physical activity in cardiac rehabilitation. *Eur J Prev Cardiolog* 2012;1):S29. doi: <http://dx.doi.org/10.1177/2047487312448007>
4. Dasgupta K, Rosenberg E, Joseph L, et al. Physician step prescription and monitoring to improve ARTERial health (SMARTER): a randomized controlled trial in patients with type 2 diabetes and hypertension. *Diabetes Obes Metab* 2017; 19(5):695-704.
5. Fayehun AF, Olowookere OO, Ogunbode AM, et al. Walking prescription of 10 000 steps per day in patients with type 2 diabetes mellitus: A randomised trial in Nigerian general practice. *Br J Gen Pract* 2018;68(667):e139-e45. doi: <http://dx.doi.org/10.3399/bjgp18X694613>
6. Grey EB, Thompson D, Gillison FB. Effects of a Web-Based, Evolutionary Mismatch-Framed Intervention Targeting Physical Activity and Diet: a Randomised Controlled Trial. Int J Behav Med. 2019 Dec;26(6):645-657. doi: 10.1007/s12529-019-09821-3.
7. *Houle J, Doyon O, Vadeboncoeur N, et al. Innovative program to increase physical activity following an acute coronary syndrome: randomized controlled trial. *Patient Educ Couns* 2011;85(3):e237-44. doi: https://dx.doi.org/10.1016/j.pec.2011.03.018
8. *Houle J, Doyon O, Vadeboncoeur N, et al. Effectiveness of a pedometer-based program using a socio-cognitive intervention on physical activity and quality of life in a setting of cardiac rehabilitation. *Can J Cardiol* 2012;28(1):27-32. doi: https://dx.doi.org/10.1016/j.cjca.2011.09.020
9. Katzmarzyk PT, Champagne CM, Tudor-Locke C, et al. A short-term physical activity randomized trial in the Lower Mississippi Delta. *PLoS ONE* 2011;6(10):e26667. doi: <https://dx.doi.org/10.1371/journal.pone.0026667>
10. Yates T, Edwardson CL, Henson J, et al. Walking Away from Type 2 diabetes: a cluster randomized controlled trial. *Diabet Med* 2017;34(5):698-707. doi: <https://dx.doi.org/10.1111/dme.13254>

*Houle 2011 and 2012 data from same study.

**References of studies with wearable tracker measuring steps-per-day but did not provide IPD**

1. Alonso-Domínguez R, Patino-Alonso MC, Sánchez-Aguadero N, García-Ortiz L, Recio-Rodríguez JI, Gómez-Marcos MA. Effect of a multifactorial intervention on the increase in physical activity in subjects with type 2 diabetes mellitus: a randomized clinical trial (EMID Study). *Eur J Cardiovasc Nurs.* 2019;18(5):399-409.
2. Chudowolska-Kiełkowska M, Małek Ł A. A nurse-led intervention to promote physical activity in sedentary older adults with cardiovascular risk factors: a randomized clinical trial (STEP-IT-UP study). *Eur J Cardiovasc Nurs.* 2020;19(7):638-645.
3. De Greef K, Deforche B, Tudor-Locke C, De Bourdeaudhuij I. A cognitive-behavioural pedometer-based group intervention on physical activity and sedentary behaviour in individuals with type 2 diabetes. *Health Educ Res.* 2010;25(5):724-736.
4. De Greef K, Deforche B, Tudor-Locke C, De Bourdeaudhuij I. Increasing physical activity in Belgian type 2 diabetes patients: a three-arm randomized controlled trial. *Int J Behav Med.* 2011;18(3):188-198.
5. De Greef KP, Deforche BI, Ruige JB, et al. The effects of a pedometer-based behavioral modification program with telephone support on physical activity and sedentary behavior in type 2 diabetes patients. *Patient Education and Counseling.* 2011;84(2):275-279.
6. Karstoft K, Winding K, Knudsen SH, et al. The effects of free-living interval-walking training on glycemic control, body composition, and physical fitness in type 2 diabetic patients: a randomized, controlled trial. *Diabetes Care.* 2013;36(2):228-236.
7. Lyons EJ, Swartz MC, Lewis ZH, Martinez E, Jennings K. Feasibility and Acceptability of a Wearable Technology Physical Activity Intervention With Telephone Counseling for Mid-Aged and Older Adults: A Randomized Controlled Pilot Trial. *JMIR Mhealth Uhealth.* 2017;5(3):e28.
8. Lystrup R, Carlsen D, Sharon DJ, Crawford P. Wearable and interactive technology to share fitness goals results in weight loss but not improved diabetes outcomes. *Obes Res Clin Pract.* 2020;14(5):443-448.
9. Martin SS, Feldman DI, Blumenthal RS, et al. mActive: A Randomized Clinical Trial of an Automated mHealth Intervention for Physical Activity Promotion. *J Am Heart Assoc.* 2015;4(11):09.
10. Paula TP, Viana LV, Neto ATZ, Leitao CB, Gross JL, Azevedo MJ. Effects of the DASH Diet and Walking on Blood Pressure in Patients With Type 2 Diabetes and Uncontrolled Hypertension: A Randomized Controlled Trial. *J Clin Hypertens (Greenwich).* 2015;17(11):895-901.
11. Piette JD, Richardson C, Himle J, et al. A randomized trial of telephonic counseling plus walking for depressed diabetes patients. *Med Care.* 2011;49(7):641-648.
12. Plotnikoff R, Karunamuni N, Courneya K, Sigal R, Johnson J, Johnson S. The Alberta Diabetes and Physical Activity Trial (ADAPT): A randomized trial evaluating theory-based interventions to increase physical activity in adults with type 2 diabetes. *Ann Behav Med.* 2013;45(1):45-56.
13. Silfee V, Petosa R, Laurent D, Schaub T, Focht B. Effect of a behavioral intervention on dimensions of self-regulation and physical activity among overweight and obese adults with type 2 diabetes: a pilot study. *Psychol Health Med.* 2016;21(6):715-723.
14. Tudor-Locke C, Bell R, Myers A, et al. Controlled outcome evaluation of the First Step Program: A daily physical activity intervention for individuals with type II diabetes. *Int J Obes (Lond).* 2004;28(1):113-119.
15. Van Dyck D, De Greef K, Deforche B, et al. The relationship between changes in steps/day and health outcomes after a pedometer-based physical activity intervention with telephone support in type 2 diabetes patients. *Health Educ Res.* 2013;28(3):539-545.
16. Yates T, Davies M, Gorely T, Bull F, Khunti K. Effectiveness of a pragmatic education program designed to promote walking activity in individuals with impaired glucose tolerance: a randomized controlled trial. *Diabetes Care.* 2009;32(8):1404-1410.

# Appendix 3: Characteristics of eligible randomised trials

**Characteristics of eligible randomised trials involving wearable trackers for measuring physical activity performance in patients with cardiometabolic conditions**

| **Study and date** | **Country** | **Data source** | **Total sample size** | **Int.** | **Con.** | **Sex (M/F)** | **Tracker type (model if specified) and length of trial** | **Cardiometabolic condition(s) focus** |
| --- | --- | --- | --- | --- | --- | --- | --- | --- |
| Alonso-Dominguez 2019 | Spain | Publication | 204 | 102 | 102 | 52/51 | Pedometer (Omron HJ-321 Triaxis), 12 months | Type II diabetes |
| Andersen 2015 | US | IPD | 38 | 18 | 20 | 24/14 | Pedometer, 3 months | Cardiac rehabilitation |
| Araiza 2006 | US | IPD | 30 | 15 | 15 | 14/16 | Pedometer (Yamax Digiwalker SW-701), 6 weeks | Newly diagnosed type II diabetes |
| Chudowolska-Kielkowska 2020 | Poland | Publication | 199 | 99 | 100 | 55/144 | Pedometer (M2 Smartband, Enwei Technology Co. Ltd., Shenzhen, China) | Cardiovascular risk factors |
| Cupples 2013 | N. Ireland | IPD | 45 | 19 | 26 | 41/4 | Pedometer (Yamax CW-701), 6 weeks | Cardiac rehabilitation |
| Dasgupta 2017 | Canada | IPD | 347 | 174 | 173 | 157/190 | Pedometer (Yamax SW-701), 12 months | Type II diabetes, hypertension |
| De Greef 2010 | Belgium | Publication | 41 | 20 | 21 | 28/13 | Pedometer (Yamax DigiWalker SW200) | Type II diabetes |
| De Greef 2011 (1) | Belgium | Publication | 67 | 43 | 24 | 47/20 | Pedometer (Yamax DigiWalker SW200) | Type II diabetes |
| De Greef 2011 (2) | Belgium | Publication | 92 | 60 | 32 | 63/29 | Pedometer (Yamax DigiWalker SW200) | Type II diabetes |
| Fayehun 2018 | Nigeria | IPD | 46 | 23 | 23 | 17/29 | Pedometer (Yamax SW-200 digi-walker), 10 weeks | Type II diabetes |
| Grey 2019 | UK | IPD | 59 | 30 | 29 | 33/26 | Pedometer (Yamax EX210 3D), 12 weeks | Adults with overweight or obesity |
| Houle 2011, 2012 | Canada | IPD | 65 | 32 | 33 | 14/51 | Pedometer (Yamax Digiwalker NL-2000), 12 months | Cardiac rehabilitation |
| Karstoft 2013 | Denmark | Publication | 32 | 24 | 8 | 20/12 | Accelerometer (Tri-axil accelerometer, JD Mate; Kissei Comtec, Matsumoto, Japan), 4 months | Type II diabetes |
| Katzmarzyk 2011 | US | IPD | 43 | 20 | 23 | 8/35 | Pedometer (Yamax Digiwalker SW-200) & ActiGraph Model GT3X, 2 weeks | Obese/Overweight |
| Lyons 2017 | US | Publication | 40 | 20 | 20 | 6/34 | Wearable electronic activity monitor (Up24, Jawbone Inc, San Francisco, CA), 12 weeks | Overweight or obesity |
| Lystrup 2020 | US | Publication | 120 | 60 | 60 | 59/49 | Fitbit Charge activity monitor, 6 months | Type II diabetes |
| Martin 2015 | US | Publication | 48 | 32 | 16 | 26/22 | Fitbug Orb (Chicago, IL), tri-axil accelerometer, 6 weeks | CVD rehabilitation |
| Paula 2015 | Brazil | Publication | 40 | 20 | 20 | 18/22 | Pedometer (Digi-Walker CW200, Yamax, Tokyo, Japan), 4 weeks | Type II diabetes |
| Piette 2011 | US | Publication | 339 | 172 | 167 | 141/150 | Pedometer (Omron HJ-720 ITC), 12 months | Type II diabetes and depressive symptoms |
| Plotnikoff 2013 | 287 | Publication | 287 | 193 | 94 | NR | Pedometer, 18 months | Type II diabetes |
| Silfee 2016 | 24 | Publication | 24 | 13 | 11 | 7/12 | Pedometer, 5 weeks | Type II diabetes and overweight or obesity |
| Tudor-Locke 2004 | Canada | Publication | 47 | 24 | 23 | 26/11 | Pedometer (Yamax DigiWalker SW200) | Type II diabetes, Obese/Overweight |
| Van Dyck 2013 | Belgium | Publication | 92 | 60 | 32 | 64/28 | Pedometer (Yamax DigiWalker SW200) | Type II diabetes |
| Yates 2009 | UK | Publication | 103 | 33 | 34 | 57/30 | Pedometer (SW-200; Yamax, Tokyo, Japan), 12 months | Overweight and obesity with impaired glucose tolerance |
| Yates 2017 | UK | IPD | 808 | 423 | 385 | 514/294 | Pedometer, 12 months | High risk of diabetes (including obesity) |

US: United States; UK: United Kingdom; IPD: individual participant data; Int. intervention; Con. Control; M: male; F: female; CVD: cardiovascular disease; NR: not reported

^µ^study had five patients missing due to Native American status and we identified two duplicate entries meaning that seven patients were missing for this dataset

**Further specifics of wearable tracker characteristics for the IPD studies included in the meta-analysis**

| **Study** | **Objective** | **Tracker (model) and Placement** | **Description of intervention/programme and outcome measurement with goals in provided** | **Behaviour change included as part of intervention design** |
| --- | --- | --- | --- | --- |
| Andersen 2015 | To compare physical activity changes between two groups of patients randomly assigned to either pedometer tracking or usual care | Pedometer (Placement: waist) | Participants who completed cardiac rehabilitation (CR) and the post-CR assessment were randomized to either a pedometer-tracking group, or a usual-care control group. Participants in the pedometer tracking group received two identical pedometers during the post-CR assessment and were instructed to wear the first pedometer during waking hours and record daily steps for seven consecutive days. After seven days, participants returned the first pedometer in a self-addressed mailing envelope and began recording daily steps with the second pedometer for the remainder of the 3 months following CR completion. | Six items created by Franks and colleagues (2006) were adapted from several functional social support measures to address social support specifically related to encouragement from family regarding healthy lifestyle behaviours.  **Measurement of behaviour:** The Seven-Day Physical Activity Recall (Blair et al., 1985) is an 8-item, self-report questionnaire that measures occupational and leisure activity during the prior seven days. It has been validated in several experimental studies and community survey samples (Blair et al., 1985). Included in the instructions are examples of moderate, hard, and very hard activities. Item responses on this measure were utilized to calculate participants’ total energy expenditure over the prior 7 days. |
| Araiza 2006 | To determine whether a recommendation to accumulate 10000 steps per day, as documented by use of a pedometer, would result in significant improvements in parameters of glycaemic control, insulin sensitivity, cardiovascular risk, lipid profile, and oxidative stress in sedentary patients with type 2 diabetes mellitus | Pedometer (Yamax Digiwalker SW-701) (Placement: waist right mid-thigh) | The control group was instructed to maintain their normal activity habits throughout the 6-week intervention. The active group was instructed to walk 10000 steps on 5 or more days of the week for 6 weeks.  Each subject wore a Yamax Digiwalker step counter (SW-701, New Lifestyles, Kansas City, MI) throughout the day, except for sleeping and bathing, and was trained regarding proper placement and use of the pedometer. The pedometers were positioned on the waist, in-line with the right mid-thigh. Each morning the pedometer was reset to zero, and each evening the subject recorded the steps accumulated during the day in an activity log. | No |
| Cupples 2013 | To explore the feasibility of a randomised controlled trial, in the context of real-world clinical practice, to determine the effectiveness of a tailored intervention using pedometer step-counts in goal setting for patients following a supervised programme of cardiac rehabilitation (CR) | Pedometer (Yamax CW-701) (Placement: unclear) | Following completion of their CR programme patients who consented were shown how to wear a validated pedometer (YAMAX Digiwalker CW-701) (Yamax Inc, Japan) and asked to wear it during waking hours, except for water-based activities, for one week. It was taped shut, obscuring step-count records, to provide a ‘blinded’ baseline measurement. In accordance with advice given during their CR programme, participants were encouraged to aim to achieve 30 minutes of moderate intensity exercise daily.  Intervention groups, based on a previous community-based study of rehabilitation for patients with back pain were asked to wear the pedometer, record daily step-counts in a diary and meet a facilitator weekly to review their progress.  **Goal:** At each meeting the facilitator checked diary records against pedometer memory values and helped individuals to set realistic step count goals for the following week, encouraging a gradual 10% increase in average daily count, aiming for 10,000 steps/day. | No (only baseline behaviour change were assessed and did not part of the PA intervention component) |
| Dasgupta 2017 | To capture impact of intervention on physical activity, but also to gauge its biological effects | Pedometer (Yamax SW-701) (Placement: waist) | Participants were typically seen by their physician in a clinical setting 3 to 4 times over a 12 to 15-month period. The control arm received advice to engage in 30 to 60 minutes of activity daily, consistent with usual care.  At the first visit, the physician received a package with a pedometer and step count log for the participant, a package of “step count prescription” scripts and the baseline steps/day as assessed during baseline evaluation.  **Goal:** The aim was to achieve a net increase over baseline of 3000 steps/day over 1 year. A step count increment of 2500 to 3000 steps is roughly equivalent to 30 minutes of walking at a moderate pace, as established through direct counts of individuals walking on a treadmill at a workload of 3 metabolic equivalents (METS)/minute | No (focus was on eating behaviour) |
| Fayehun 2018 | To examine the effect of a 10,000 steps per day prescription on glycaemic control of patients with T2DM | Pedometer (Yamax SW-200 digi-walker) (Placement: waist-mounted) | **Goal:** The intervention group participants were given the goal of accumulating 10 000 steps per day during the following 10-week intervention period. They were counselled to increase their daily step count by 20% from baseline each week, until the 10 000 steps goal was reached.  Possible motivators and barriers to walking were identified. Additional counselling was given at weeks 4 and 8 visit, and telephone follow-up at weeks 2, 6, and 10. Control group participants were asked to maintain their normal activity habits and encouraged to keep daily step count during follow-up. At week 11 visit, baseline measurements were repeated. All measurements and counselling were done by the authors, who were not blinded to the treatment group. | No |
| Grey 2019 | To test the effectiveness of a 12-week, novel online intervention (Evolife) aiming to increase  physical activity level and reduce energy intake among overweight/obese adults | Pedometer (BodyMedia SenseWear Core and pedometer which were based around a ‘Evolife’ website) | The intervention (Evolife) was based around a website that aimed to provide participants with information, framed from an evolutionary mismatch perspective, about physical activity and healthy eating, and advice on how to make behavioural changes to improve health.  **Goal:** The goal for physical activity was a daily step goal which was measured with the pedometer. Each individual set their own step goal during the trial. | Evolife intervention provide advice on how to improve and change behaviours. Specifically for pedometer use the BCT Taxonomy v.1 2013 was used for self-monitoring of steps a day. |
| Houle 2011-2012 | To evaluate the impact of a socio-cognitive intervention associated with a pedometer-based program on physical activity, cardiovascular risk factors, quality of life and self-efficacy expectation during one year following an acute coronary syndrome. | Pedometer (Yamax Digiwalker NL-2000) (Placement: waist) | Participants in the intervention group were given a pedometer (Yamax Digiwalker SW-200, Lees Summit, USA), diary and information regarding PA after an acute coronary syndrome. Clinical nurse specialist provided instructions (such as: how to wear pedometer correctly in waistband, how to perform exercise safety, etc.) and recommended exercise goal (example: walking 3000 steps in 30 min). Walking at a moderate intensity according to the Borg Scale was encouraged. Pedometer and diary were used to allow daily self-monitoring of PA. Type, duration, intensity of exercise sessions and personal feedback were recorded in a diary.    **Goal:** The subjects were asked to set a target of 3000 steps per day increment in physical activity. | The social cognitive theory (Bandura 1986) was the theoretical framework used for the clinical nurse specialist intervention. This theory suggests conditions of health promoting interactions and four sources of efficacy expectation.  Includes the four components: 1) verbal persuasion; 2) physiological states; 3) Vicarious experience; 4) performance accomplishments |
| Katzmarzyk 2011 | To determine if a short-term pedometer-based intervention results in immediate increases in time spent in moderate-to-vigorous physical activity (MVPA) compared to a minimal educational intervention. | Pedometer (Yamax Digiwalker SW-200) was part of intervention, but the ActiGraph Model GT3X accelerometer was used to measure the primary outcome of MVPA (Placement: waist (right-hip) | The education plus pedometer group received the same educational materials, in addition to a YAMAX Digi-Walker SW-200 pedometer and instructions on its use. The participants were shown how to operate the pedometer and walked outside with an interventionist for approximately 10 minutes to build self-efficacy for walking at MVPA and to observe how quickly steps accrued. | No |
| Yates 2017 | To investigate whether an established behavioural intervention, Walking Away from Type 2 Diabetes, is effective at promoting and sustaining increased walking activity when delivered within primary care | Programme included a pedometer (A pedometer and step/day diary were provided free), but accelerometer (GT3X Actigraph) was used to measure ambulatory activity as secondary outcome (Placement: Waist - placed on right anterior axillary) | The intervention group were offered the 3-h Walking Away from Type 2 Diabetes Mellitus group-based structured educational programme (henceforth referred to as Walking Away), described in detail previously.  Physical activity was promoted by targeting self-efficacy, identifying barriers and promoting self-regulatory skills through pedometer use.  **Goal:** Individuals were encouraged to increase their physical activity levels up to 3000 step/day over baseline levels depending on individual preference and ability. Goal attainment was encouraged through the use of smaller proximal objectives, such as increasing activity by 500 steps/day every fortnight. Participants set an action plan detailing where, when, and how their first proximal goal would be reached and were encouraged to repeat this process for each new goal. A pedometer and step/day diary were provided free. | No (behaviour change element of intervention focused on type II diabetes, generally) |

# Appendix 4: Assessment of risk of bias

**Assessment of risk of bias in all eligible studies measuring steps-per-day (n=25), studies with IPD (n=9), and studies without access to IPD (n=16)**

The original study RoB classifications of all studies by each domain are available at: Hodkinson A, Kontopantelis E, Adeniji C, van Marwijk H, McMillian B, Bower P, Panagioti M. Interventions Using Wearable Physical Activity Trackers Among Adults With Cardiometabolic Conditions: A Systematic Review and Meta-analysis. JAMA Netw Open. 2021 Jul 1;4(7):e2116382. doi: 10.1001/jamanetworkopen.2021.16382. PMID: 34283229.

**Risk of bias assessment of each of the IPD studies by each domain**

| **Study** | **Random sequence generation** | **Allocation concealment** | **Blinding of outcome assessment*** | **Incomplete outcome data** | **Selective reporting bias** |
| --- | --- | --- | --- | --- | --- |
| Andersen 2015 | Unclear | Unclear | High | Low | Low |
| Araiza 2006 | Unclear | Unclear | Low | Unclear | Low |
| Cupples 2013 | Low | Low | Low | Low | Low |
| Dasgupta 2017 | Low | Unclear | Low | Low | Low |
| Fayehun 2018 | Low | Low | Unclear | High | Unclear |
| Grey 2017 | Unclear | Low | High | Low | Low |
| Houle 2011, 2012 | Low | Unclear | Low | Low | Low |
| Katzmarzyk 2011 | Low | Low | Low | Low | Low |
| Yates 2017 | Low | Low | Low | Low | Low |

*Blinding of patient and provider was not assessed since both could see the device and so concealment was not necessary.

# Appendix 5: Assessment of publication bias

**IPD funnel plot of wearable trackers measuring steps-per-day**

**Egger's test for small-study effects:**

Regress standard normal deviate of intervention

effect estimate against its standard error

Number of studies = 9 Root MSE = 1.154

------------------------------------------------------------------------------

Std_Eff | Coef. Std. Err. t P>|t| [95% Conf. Interval]

-------------+----------------------------------------------------------------

slope | 31.83001 178.539 0.18 0.864 -390.3476 454.0076

bias | 2.612177 .5570916 4.69 **0.002** 1.294865 3.92949

------------------------------------------------------------------------------

Test of H0: no small-study effects P = 0.002

**Funnel plot of IPD and AD together measuring steps-per-day with the wearable trackers**

**Egger's test for small-study effects:**

Regress standard normal deviate of intervention

effect estimate against its standard error

Number of studies = 24 Root MSE = 3.812

------------------------------------------------------------------------------

Std_Eff | Coef. Std. Err. t P>|t| [95% Conf. Interval]

-------------+----------------------------------------------------------------

slope | 540.6436 416.9367 1.30 0.208 -324.0302 1405.317

bias | 3.241834 1.187443 2.73 **0.012** .7792286 5.70444

------------------------------------------------------------------------------

Test of H0: no small-study effects P = 0.012

**Funnel plot of low risk of bias IPD studies vs. high risk of bias IPD studies (based on allocation concealment)**

1: High risk of bias studies (based on allocation concealment judged at either unclear or high risk); 2: low risk of bias studies

**Egger’s test for low risk of bias studies:**

Regress standard normal deviate of intervention

effect estimate against its standard error

Number of studies = 6 Root MSE = 1.281

------------------------------------------------------------------------------

Std_Eff | Coef. Std. Err. t P>|t| [95% Conf. Interval]

-------------+----------------------------------------------------------------

slope | 121.2811 215.5527 0.56 0.604 -477.189 719.7513

bias | 2.054678 .8077134 2.54 0.064 -.1878939 4.29725

------------------------------------------------------------------------------

Test of H0: no small-study effects P = 0.064

**Egger’s test for high risk of bias studies:**

Regress standard normal deviate of intervention

effect estimate against its standard error

Number of studies = 3 Root MSE = .4279

------------------------------------------------------------------------------

Std_Eff | Coef. Std. Err. t P>|t| [95% Conf. Interval]

-------------+----------------------------------------------------------------

slope | 6923.744 3653.248 1.90 0.309 -39495.18 53342.66

bias | -4.081342 3.876251 -1.05 0.484 -53.33378 45.1711

------------------------------------------------------------------------------

Test of H0: no small-study effects P = 0.484

# Appendix 6: Secondary outcome IPD-MAs results

**Effects of wearable tracker interventions on the secondary outcomes summarised using individual participant data (IPD)**

| **Secondary outcomes** | **No. of studies (no. of participants)** | **Intervention (mean: SD)** | **Control (mean: SD)** | **Standardised mean differences (95% CIs, I^2^ %)** | **Mean difference (95% CIs, I^2^ %)** |
| --- | --- | --- | --- | --- | --- |
| Glucose (hbA1c %) | 5 (1290) | 6.25 (1.27) | 6.22 (1.17) | - | -0.13 (-0.30, 0.03), 2.6% |
| Glucose (mmols/mol) | 5 (1290) | 44.8 | 44.5 | - | - |
| Blood pressure: |  |  |  |  |  |
| - SBP (mmHg) | 6 (1363) | 128.67 (16.18) | 131.26 (18.44) | - | -0.70 (-2.84, 1.44), 1.2% |
| - DBP (mmHg) | 6 (1363) | 80.17 (10.55) | 81.22 (11.20) | - | -0.33 (-1.17, 0.51), 0.0% |
| Cholesterol: |  |  |  |  |  |
| - Total (mmols/mol) | 4 (1244) | 9.31 (29.99) | 9.57 (30.07) | 1.76 (-2.02, 5.53), 1.4% | - |
| - HDL (mmols/mol) | 5 (1309) | 2.32 (6.64) | 2.38 (6.74) | 0.02 (-0.14, 0.18), 0.0% | - |
| - LDL (mmols/mol) | 5 (1309) | 5.15 (17.03) | 5.22 (16.52) | 1.18 (-1.38, 3.74), 5.7% | - |
| BMI (kg/m2) | 5 (1295) | 31.83 (5.48) | 31.82 (5.02) | - | 0.03 (-0.17, 0.24), 0.0% |
| Weight (kg) | 4 (1303) | 89.25 (17.89) | 88.96 (16.04) | - | 0.03 (-0.93, 0.98), 0.0% |

No. Number; SD: standard deviation; CIs: confidence intervals; hbA1c: haemoglobin A1c test; SBP: systolic blood pressure; DBP: diastolic blood pressure; HDL: high-density lipoproteins; LDL: low-density lipoproteins; BMI: body mass index.

# Appendix 7: Two stage IPD meta-analysis

**Two stage IPD-MAs of wearable trackers measuring steps-per-day**

# Appendix 8: Intervention and programme subgroup factors and other sensitivity analysis

**Differential effects of wearable trackers for steps-per-day performance among subgroups of intervention factors and other important sensitivity analysis**

|  |  |  | **Intervention covariate interaction** | | |
| --- | --- | --- | --- | --- | --- |
| **Characteristic** | **No. of studies** | **Mean difference* (95% CI)** | **Coefficient; 95% CI** | **P-value** | **I^2^ (95% CI) (%)** |
| Social cognitive theory used: |  |  |  |  |  |
| - Not part of programme | 6 | 1422.02 (633.55, 2210.50) | 1 | NA | 13.20 (3.1, 41.7) |
| - Part of programme | 3 | 2476.31 (935.28, 4017.34) | -1054 (-2785.38, 676.80) | 0.233 |  |
| Goal used: |  |  |  |  |  |
| - No | 3 | 2218.45 (834.05, 3602.85) | 1 | NA | 14.7 (3.9, 42.4) |
| - Yes | 6 | 1433.49 (566.35, 2300.62) | 784.96 (-848.61, 2418.54) | 0.346 |  |
| Placement of intervention: |  |  |  |  |  |
| - Wrist | 1 | 1960.43 (-494.03, 4414.89) | 1 | NA | 16.7 (4.8, 44.6) |
| - Waist | 8 | 1637.86 (816.69, 2459.03) | 322.57 (-2265.68, 2910.82) | 0.807 |  |
| Performance over time: |  |  |  |  |  |
| - < 26 weeks | 6 | 1999.90 (1068.12, 2931.67) | 1 | NA | 13.74 (3.3, 42.4) |
| - ≥ 26 weeks | 3 | 1143.33 (32.68, 2253.97) | -856.57 (-2306.32, 593.17) | 0.247 |  |
| Low risk of bias |  |  |  |  |  |
| - No | 3 | 1293.76 (-10.86, 2598.39) | 1 | NA | 16.4 (4.8, 43.1) |
| - Yes | 6 | 1868.51 (912.83, 2824.20) | 574.75 (-1042.48, 2191.98) | 0.486 |  |
| Delivery format |  |  |  |  |  |
| - Self-managed | 5 | 850.31 (325.30, 1375.32) | 1 | NA | 3.20 (0.3, 28.8) |
| - Face-to-face | 4 | 2629.84 (1834.68, 3425.00) | 1779.53 (826.54, 2732.51) | **<0.0001** |  |

*Model accounted for baseline physical activity scores.

# Appendix 9: Studies not contributing IPD

**Meta-analysis for wearable tracker studies for measuring steps-per-day which did not provide IPD [MD]**

# Appendix 10: List of collaborators

**Anderson 2015**

Professor Charles Emery

Department of Psychology, The Ohio State University College of Arts and Sciences, 145 Psychology Building, 1835 Neil Ave. Columbus, OH. 43210

**Araiza 2006**

Dr Mark R. Burge

Department of Medicine, Endocrinology and Metabolism, University of New Mexico Health Sciences Center, MSC105500, Albuquerque, NM 87131, USA

Mr Hunter Esmiol

Department of Medicine, Endocrinology and Metabolism, University of New Mexico Health Sciences Center, MSC105500, Albuquerque, NM 87131, USA

**Cupples 2013**

Professor Margaret E. Cupples

Department of General Practice and Primary Care, Centre for Public Heath, Queen’s University Belfast, Institute for Clinical Science, Belfast, BT12 6BA, UK

Professor Mark A. Tully

School of Health Sciences, Institute of Mental Health Sciences, Ulster University Shore Road, Newtownabbey, Co. Antrim, BT37 0QB, UK

**Dasgupta 2017**

Professor Kaberi Dasgupta

Department of Medicine, McGill University Health Centre, Montréal, QC, Canada

Centre for Outcomes Research and Evaluation (CORE), Research Institute of the McGill University Health Centre, 5252 Boulevard de Maisonneuve O, Montréal, QC, H4A 3S9, Canada

Professor Stella S. Daskalopoulou

Department of Medicine, Division of Internal Medicine and Division of Experimental Medicine.

McGill University Health Centre, 1001 Decarie Boulevard, Montreal, QC, H4A 3J1, Canada

Dr Alexandra B. Cooke

Division of Experimental Medicine, Department of Medicine, McGill University, 1001 Decarie Boulevard, Montreal, QC, H4A 3J1, Canada

**Fayehun 2018**

Dr Ayorinde Fasina Fayehun

Department of Family Medicine, University College Hospital, Queen Elizabeth Road, Ibadan, Nigeria.

**Grey 2019**

Dr Elisabeth Grey

Department for health, University of Bath, Bath, BA2 7AY, United Kingdom.

**Houle 2011, 2012**

Professor Julie Houle

[Department of Nursing, Université du Québec à Trois-Rivières, 3351, boul. des Forges, Trois-Rivières (Québec) G8Z 4M3,Canada.](https://www.researchgate.net/institution/Universite_du_Quebec_a_Trois-Rivieres)

Dr Paul Poirier

Institut Universitaire de Cardiologie et de Pneumologie de Québec, Centre de Recherche Clinique, 2725 chemin Ste-Foy, Québec, G1V 4G5, Canada

**Katzmarzyk 2011**

Professor Peter Katzmarzyk

Population Science, Pennington Biomedical Research Centre, Baton Rouge, Louisiana, United States of America

**Yates 2017**

Professor Thomas Yates

Diabetes Research Centre, University of Leicester, Leicester General Hospital, Gwendolen Road, Leicester, LE5 4PW

NIHR Leicester Biomedical Research Centre, University of Leicester, LE1 7RH

Dr Joseph Henson

Diabetes Research Centre, University of Leicester, Leicester General Hospital, Gwendolen Road, Leicester, LE5 4PW

NIHR Leicester Biomedical Research Centre, University of Leicester, LE1 7RH

# Appendix 11: IPD PRISMA checklist

**PRISMA-IPD Checklist of items to include when reporting a systematic review and meta-analysis of individual participant data (IPD)**

| **PRISMA-IPD**  **Section/topic** | **Item No** | **Checklist item** | **Reported on page** |
| --- | --- | --- | --- |
| **Title** | | | |
| Title | 1 | Identify the report as a systematic review and meta-analysis of individual participant data. | 1 |
| **Abstract** | | | |
| Structured summary | 2 | Provide a structured summary including as applicable: | 3 |
|  |  | **Background**: state research question and main objectives, with information on participants, interventions, comparators and outcomes. |  |
|  |  | **Methods**: report eligibility criteria; data sources including dates of last bibliographic search or elicitation, noting that IPD were sought; methods of assessing risk of bias. |  |
|  |  | **Results**: provide number and type of studies and participants identified and number (%) obtained; summary effect estimates for main outcomes (benefits and harms) with confidence intervals and measures of statistical heterogeneity. Describe the direction and size of summary effects in terms meaningful to those who would put findings into practice. |  |
|  |  | **Discussion:** state main strengths and limitations of the evidence, general interpretation of the results and any important implications. |  |
|  |  | **Other:** report primary funding source, registration number and registry name for the systematic review and IPD meta-analysis. |  |
| **Introduction** | | | |
| Rationale | 3 | Describe the rationale for the review in the context of what is already known. | 4 |
| Objectives | 4 | Provide an explicit statement of the questions being addressed with reference, as applicable, to participants, interventions, comparisons, outcomes and study design (PICOS). Include any hypotheses that relate to particular types of participant-level subgroups. | 5 |
| **Methods** | | | |
| Protocol and registration | 5 | Indicate if a protocol exists and where it can be accessed. If available, provide registration information including registration number and registry name. Provide publication details, if applicable. | 5 |
| Eligibility criteria | 6 | Specify inclusion and exclusion criteria including those relating to participants, interventions, comparisons, outcomes, study design and characteristics (e.g. years when conducted, required minimum follow-up). Note whether these were applied at the study or individual level i.e. whether eligible participants were included (and ineligible participants excluded) from a study that included a wider population than specified by the review inclusion criteria. The rationale for criteria should be stated. | 5-6 |
| Identifying studies - information sources | 7 | Describe all methods of identifying published and unpublished studies including, as applicable: which bibliographic databases were searched with dates of coverage; details of any hand searching including of conference proceedings; use of study registers and agency or company databases; contact with the original research team and experts in the field; open adverts and surveys. Give the date of last search or elicitation. | 5 |
| Identifying studies - search | 8 | Present the full electronic search strategy for at least one database, including any limits used, such that it could be repeated. | 5 |
| Study selection processes | 9 | State the process for determining which studies were eligible for inclusion. | 5-6 |
| Data collection processes | 10 | Describe how IPD were requested, collected and managed, including any processes for querying and confirming data with investigators. If IPD were not sought from any eligible study, the reason for this should be stated (for each such study). | 6-7 |
|  |  | If applicable, describe how any studies for which IPD were not available were dealt with. This should include whether, how and what aggregate data were sought or extracted from study reports and publications (such as extracting data independently in duplicate) and any processes for obtaining and confirming these data with investigators. |  |
| Data items | 11 | Describe how the information and variables to be collected were chosen. List and define all study level and participant level data that were sought, including baseline and follow-up information. If applicable, describe methods of standardising or translating variables within the IPD datasets to ensure common scales or measurements across studies. | 6-7 |
| IPD integrity | A1 | Describe what aspects of IPD were subject to data checking (such as sequence generation, data consistency and completeness, baseline imbalance) and how this was done. | 6-7 |
| Risk of bias assessment in individual studies. | 12 | Describe methods used to assess risk of bias in the individual studies and whether this was applied separately for each outcome. If applicable, describe how findings of IPD checking were used to inform the assessment. Report if and how risk of bias assessment was used in any data synthesis. | 6 |
| Specification of outcomes and effect measures | 13 | State all treatment comparisons of interests. State all outcomes addressed and define them in detail. State whether they were pre-specified for the review and, if applicable, whether they were primary/main or secondary/additional outcomes. Give the principal measures of effect (such as risk ratio, hazard ratio, difference in means) used for each outcome. | 6-7 |
| Synthesis methods | 14 | Describe the meta-analysis methods used to synthesise IPD. Specify any statistical methods and models used. Issues should include (but are not restricted to):   - Use of a one-stage or two-stage approach. - How effect estimates were generated separately within each study and combined across studies (where applicable). - Specification of one-stage models (where applicable) including how clustering of patients within studies was accounted for. - Use of fixed or random effects models and any other model assumptions, such as proportional hazards. - How (summary) survival curves were generated (where applicable). - Methods for quantifying statistical heterogeneity (such as I^2^ and τ^2^). - How studies providing IPD and not providing IPD were analysed together (where applicable). - How missing data within the IPD were dealt with (where applicable). | 7 |
| Exploration of variation in effects | A2 | If applicable, describe any methods used to explore variation in effects by study or participant level characteristics (such as estimation of interactions between effect and covariates). State all participant-level characteristics that were analysed as potential effect modifiers, and whether these were pre-specified. | 7 |
| Risk of bias across studies | 15 | Specify any assessment of risk of bias relating to the accumulated body of evidence, including any pertaining to not obtaining IPD for particular studies, outcomes or other variables. | 7-8 |
| Additional analyses | 16 | Describe methods of any additional analyses, including sensitivity analyses. State which of these were pre-specified. | 7-8 |
| **Results** | | | |
| Study selection and IPD obtained | 17 | Give numbers of studies screened, assessed for eligibility, and included in the systematic review with reasons for exclusions at each stage. Indicate the number of studies and participants for which IPD were sought and for which IPD were obtained. For those studies where IPD were not available, give the numbers of studies and participants for which aggregate data were available. Report reasons for non-availability of IPD. Include a flow diagram. | 8 |
| Study characteristics | 18 | For each study, present information on key study and participant characteristics (such as description of interventions, numbers of participants, demographic data, unavailability of outcomes, funding source, and if applicable duration of follow-up). Provide (main) citations for each study. Where applicable, also report similar study characteristics for any studies not providing IPD. | 8 |
| IPD integrity | A3 | Report any important issues identified in checking IPD or state that there were none. | 8 |
| Risk of bias within studies | 19 | Present data on risk of bias assessments. If applicable, describe whether data checking led to the up-weighting or down-weighting of these assessments. Consider how any potential bias impacts on the robustness of meta-analysis conclusions. | 8-9 |
| Results of individual studies | 20 | For each comparison and for each main outcome (benefit or harm), for each individual study report the number of eligible participants for which data were obtained and show simple summary data for each intervention group (including, where applicable, the number of events), effect estimates and confidence intervals. These may be tabulated or included on a forest plot. | 9 |
| Results of syntheses | 21 | Present summary effects for each meta-analysis undertaken, including confidence intervals and measures of statistical heterogeneity. State whether the analysis was pre-specified, and report the numbers of studies and participants and, where applicable, the number of events on which it is based. | 9-10 |
|  |  | When exploring variation in effects due to patient or study characteristics, present summary interaction estimates for each characteristic examined, including confidence intervals and measures of statistical heterogeneity. State whether the analysis was pre-specified. State whether any interaction is consistent across trials. |  |
|  |  | Provide a description of the direction and size of effect in terms meaningful to those who would put findings into practice. |  |
| Risk of bias across studies | 22 | Present results of any assessment of risk of bias relating to the accumulated body of evidence, including any pertaining to the availability and representativeness of available studies, outcomes or other variables. | 9 |
| Additional analyses | 23 | Give results of any additional analyses (e.g. sensitivity analyses). If applicable, this should also include any analyses that incorporate aggregate data for studies that do not have IPD. If applicable, summarise the main meta-analysis results following the inclusion or exclusion of studies for which IPD were not available. | 9-10 |
| **Discussion** | | | |
| Summary of evidence | 24 | Summarise the main findings, including the strength of evidence for each main outcome. | 10-11 |
| Strengths and limitations | 25 | Discuss any important strengths and limitations of the evidence including the benefits of access to IPD and any limitations arising from IPD that were not available. | 11 |
| Conclusions | 26 | Provide a general interpretation of the findings in the context of other evidence. | 12 |
| Implications | A4 | Consider relevance to key groups (such as policy makers, service providers and service users). Consider implications for future research. | 12 |
| **Funding** | | | |
| Funding | 27 | Describe sources of funding and other support (such as supply of IPD), and the role in the systematic review of those providing such support. | 8 |

**A1 – A3 denote new items that are additional to standard PRISMA items. A4 has been created as a result of re-arranging content of the standard PRISMA statement to suit the way that systematic review IPD meta-analyses are reported.**

© Reproduced with permission of the PRISMA IPD Group, which encourages sharing and reuse for non-commercial purposes.
